# The Roles and Acting Mechanism of *Caenorhabditis elegans* DNase II Genes in Apoptotic DNA Degradation and Development

**DOI:** 10.1371/journal.pone.0007348

**Published:** 2009-10-07

**Authors:** Huey-Jen Lai, Szecheng J. Lo, Eriko Kage-Nakadai, Shohei Mitani, Ding Xue

**Affiliations:** 1 Department of Molecular, Cellular and Developmental Biology, University of Colorado, Boulder, Colorado, United States of America; 2 Division of Microbiology, Graduate Institute of Biomedical Sciences, Chang Gung University, Taoyuan, Taiwan; 3 Department of Life Science, Chang Gung University, Taoyuan, Taiwan; 4 Department of Physiology, Tokyo Women's Medical University, School of Medicine and CREST, Japan Science and Technology, Tokyo, Japan; University of Washington, United States of America

## Abstract

DNase II enzymes are acidic endonucleases that have been implicated in mediating apoptotic DNA degradation, a critical cell death execution event. *C. elegans* genome contains three DNase II homologues, NUC-1, CRN-6, and CRN-7, but their expression patterns, acting sites, and roles in apoptotic DNA degradation and development are unclear. We have conducted a comprehensive analysis of three *C. elegans* DNase II genes and found that *nuc-1* plays a major role, *crn-6* plays an auxiliary role, and *crn-7* plays a negligible role in resolving 3′ OH DNA breaks generated in apoptotic cells. Promoter swapping experiments suggest that *crn-6* but not *crn-7* can partially substitute for *nuc-1* in mediating apoptotic DNA degradation and both fail to replace *nuc-1* in degrading bacterial DNA in intestine. Despite of their restricted and largely non-overlapping expression patterns, both CRN-6 and NUC-1 can mediate apoptotic DNA degradation in many cells, suggesting that they are likely secreted nucleases that are retaken up by other cells to exert DNA degradation functions. Removal or disruption of NUC-1 secretion signal eliminates NUC-1's ability to mediate DNA degradation across its expression border. Furthermore, blocking cell corpse engulfment does not affect apoptotic DNA degradation mediated by *nuc-1*, suggesting that NUC-1 acts in apoptotic cells rather than in phagocytes to resolve 3′ OH DNA breaks. Our study illustrates how multiple DNase II nucleases play differential roles in apoptotic DNA degradation and development and reveals an unexpected mode of DNase II action in mediating DNA degradation.

## Introduction

Programmed cell death, or apoptosis, is a conserved cellular process critical for animal development and tissue homeostasis. During apoptosis, a series of morphological changes occur due to the activation of the cell killing caspases, including chromatin condensation and fragmentation.[1], [2] The cleavage of chromatin into nucleosomal fragments is a hallmark of apoptosis and has been shown to promote cell killing [1], [3].

Multiple nucleases have been identified to catalyze fragmentation of chromosomal DNA during apoptosis. DFF40 (40 kDa DNA fragmentation factor) or CAD (caspase-activated DNase) plays a major role in generating internucleosomal DNA ladders during mammalian apoptosis [4], [5]. DFF40/CAD is kept in check in normal cells by its cognate inhibitor DFF45 (45 kDa DNA fragmentation factor) or ICAD (inhibitor of CAD) but unleashed to fragment chromosomal DNA when DFF45 is cleaved and inactivated by caspases such as caspase-3 during apoptosis [6]. DFF40 acts in a neutral pH environment but needs Mg^2+^ for optimal activity to generate DNA fragments with 3′ hydroxyl ends (3′OH) [7]. Apoptotic cells from mice deficient in DFF45/ICAD or CAD are resistant to DNA fragmentation [8], [9]. However, DNA degradation still occurs after phagocytosis of CAD −/− apoptotic cells by macrophages from wild type mice but not from deoxyribonuclease II (DNase II) deficient mice, suggesting that DNase II in phagocytes can contribute to degradation of chromosomal DNA in apoptotic cells [10].

In contrast to DFF40/CAD, DNase II is Ca^2+^/Mg^2+^-independent and has maximum activity at acidic conditions (pH 5.0–6.0) [11]. During apoptosis, apoptotic cells are acidified, which may activate acidic DNases including DNase II to degrade chromosome DNA [11], [12]. DNase II is known to digest double stranded DNA to generate 3′ phosphate and 5′ OH DNA breaks [11], [13]–[16]. Analysis of the DNase II expression patterns in mammals reveals that DNase IIα is ubiquitously expressed, whereas DNase IIβ mRNA is highly expressed in the salivary gland, eye lens and liver but is expressed at low levels in other tissues [16]–[19]. Mice deficient in DNase IIα die before birth due to anemia, indicating that DNase IIα is required for definitive erythropoiesis [20]. DNase IIβ knockout mice develop cataracts of the nucleus lentis, which is caused by undigested nuclear DNA during lens cell differentiation [21]. Therefore, DNase II nucleases are also important for animal development.

In *C. elegans*, no DFF40/CAD or DFF45/ICAD homologues have been identified [3]. However, NUC-1, a DNase II homologue, and CPS-6, a homologue of the human mitochondrial endonuclease G [22], are found to be important for apoptotic DNA degradation [23], [24]. In *nuc-1* and *cps-6* loss-of-function (*lf*) mutants, increased numbers of apoptotic cells are stained by TUNEL, which specifically labels 3′ OH DNA breaks, suggesting that NUC-1 and CPS-6 play a role in resolving DNA fragments with 3′ OH ends in apoptotic cells. Interestingly, loss of *cps-6* delays progression of apoptosis and can even block cell death in sensitized genetic backgrounds [24], whereas loss of *nuc-1* does not seem to affect cell killing or the kinetics of cell death [23], [24]. These observations suggest that *nuc-1* and *cps-6* play different roles in apoptosis, with *cps-6* acting early during apoptosis and *nuc-1* functioning at a later stage of apoptosis. Moreover, in an RNAi-based functional genomic screen, seven additional cell death-related nucleases (CRNs) have been identified [25]. Most of these CRN nucleases affect normal progression of apoptosis and some appear to form a DNA degradation complex (degradeosome) with CPS-6 to promote apoptotic DNA degradation [25].

In addition to NUC-1, there are two other DNase II homologues in *C. elegans*, CRN-6 (K04H4.6) and CRN-7 (F09G8.2). *crn-6(RNAi)* causes increased number of TUNEL-positive cells in wild-type *C. elegans* embryos as well as in *nuc-1(lf)* embryos, suggesting that CRN-6 acts in parallel to NUC-1 to promote apoptotic DNA degradation [25]. Unlike *cps-6* and other *crn* genes, *crn-6* does not affect cell killing or the kinetics of apoptosis and may act at a later stage of apoptosis like *nuc-1* [25]. The role of *crn-7* in apoptotic DNA degradation is unclear. In addition to its role in apoptosis, *nuc-1* plays a role in degrading DNA of ingested bacteria in the intestinal lumen [23], [26].

To investigate the roles of three DNase II genes in *C. elegans* apoptosis and development, we analyze their expression patterns, their mutant phenotypes, their activities in mediating DNA degradation in promoter swapping experiments, and the importance of their secretion to their DNA degradation functions. Our results indicate that three *C. elegans* DNase II genes play differential roles in *C. elegans* apoptosis and development and provide unique mechanistic insights into how DNase II nucleases mediate apoptotic DNA degradation.

## Results

### *nuc-1, crn-6* and *crn-7* affect apoptotic DNA degradation but do not affect the activation or the kinetics of apoptosis

Chromosomal DNA fragments generated during apoptosis often contain 3′ OH ends that can be labeled by the terminal deoxynucleotidyl transferase dUTP nick end labeling (TUNEL) technique [27]–[29]. Since DNase II generates DNA fragments with 3′ phosphate ends that are not labeled by TUNEL [11], [13]–[16], DNase II may play a role in resolving TUNEL-positive DNA ends generated by upstream nuclease(s). Indeed, TUNEL-positive signals accumulate in the *nuc-1*(*lf*) mutants as well as in *crn-6(RNAi)* animals [23], [25], suggesting that NUC-1 and CRN-6, two DNase II homologues, are involved in resolving TUNEL-positive DNA ends. Sequence alignment of NUC-1, CRN-6 and CRN-7, the third worm DNase II homologue, with human DNase IIα and DNase IIβ reveals an overall sequence similarity, including the identical residues around the active site Histidine (Figure S1A). In addition, all these proteins contain a secretion signal at the amino terminus based on the SignaIP program analysis.

**Figure 1:**
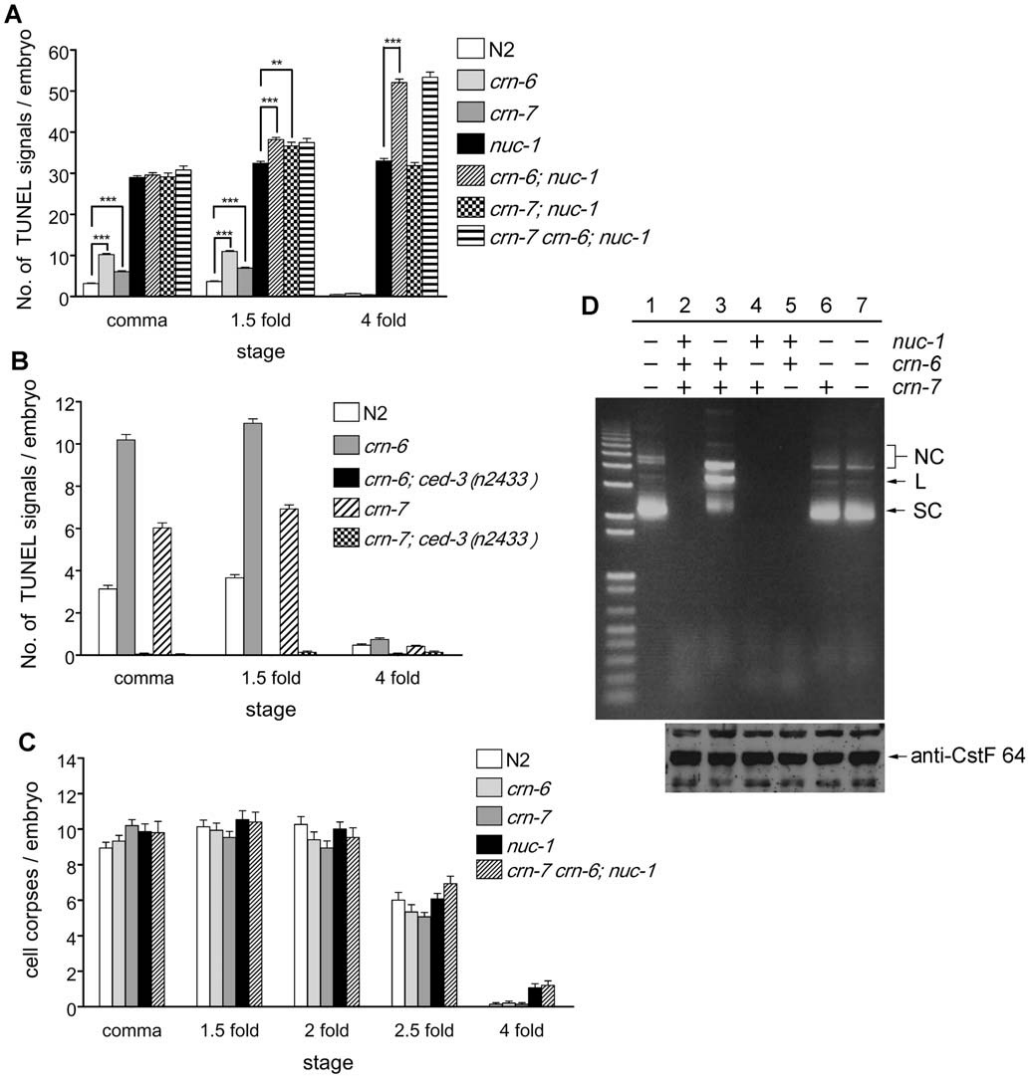
Analyses of animals deficient in three *C. elegans* DNase II genes. A and B) TUNEL staining. TUNEL signals were scored in comma, 1.5-fold, and 4-fold stage embryos of the indicated genotypes. At least 20 embryos at each embryonic stage were scored. Error bars indicate standard error of mean (SEM). The significance of differences in two different genetic backgrounds indicated by brackets was determined by student *t* test. ** *P*<0.01, *** *P*<0.001. C) Cell corpse analysis. The numbers of cell corpses in embryos of the indicated genotypes at five different embryonic stages were scored. 15 embryos were counted for each embryonic stage. Error bars indicate SEM. In A–C, *crn-6(tm890)*, *crn-7(ok866)*, and *nuc-1(e1392)* were the alleles used. D) Acidic DNase activity assay. Upper panel: 1 µg of worm lysate were incubated with 750 ng of pSL1190 plasmid DNA at 37°C for 10 min (in 50 mM acetic acid, pH 5.0)(see Materials and Methods). Lane 1, no lysate; lane 2, lysate from N2 animals; lane 3, lysate from *nuc-1(e1392)* animals; lane 4, lysate from *crn-6(tm890)* animals; lane 5, lysate from *crn-7(ok866)* animals; lane 6, lysate from *crn-6(tm890); nuc-1(e1392)* animals; lane 7, lysate from *crn-7(ok866) crn-6(tm890); nuc-1(e1392)* animals. SC, L and NC indicate the supercoiled, linear and nicked circular form of plasmid DNA, respectively. Lower Panel: a western blot analysis using anti-CstF-64 antibody reveals that similar amounts of lysate were loaded for the DNase activity assay shown above. Arrow indicates the CstF-64 protein band.

To understand the functions of three *C. elegans* DNase II homolgues, we analyzed the loss-of-function phenotypes of these genes. We isolated an 843 bp deletion mutation (*tm890)* in *crn-6* that removes exons 3–6 of *crn-6* and is likely a null allele. We also obtained a 527 bp deletion (*ok866*) in *crn-*7 that removes one third of exon 2 and almost all intron 2 (Figure S1B). For *nuc-1*, the *e1392* strong loss-of-function mutation that results in an early stop codon at amino acid 59 was used [23]. TUNEL assays were performed on wild type (N2) embryos as well as on *nuc-1*, *crn-6* and *crn-7* mutant embryos (see Materials and Methods). *nuc-1(e1392)*, *crn-6(tm890)* and *crn-7(ok866)* mutant embryos all had more TUNEL-positive signals than N2 embryos (Figure 1A). Compared with wild type embryos that had an average 3.1, 3.7 and 0.5 TUNEL-positive cells at the comma, 1.5-fold, and 4-fold stages, respectively, the *nuc-1(e1392)* mutant embryos had approximately 30 TUNEL-positive signals at these three embryonic stages (Figure 1A)[23]. Since few cells die in late embryonic stages (3-fold and 4-fold stages), the TUNEL-reactive signals seen in 4-fold *nuc-1* mutant embryos must be DNA breaks that were generated during early embryonic stages and persisted through the late embryonic stages [23], [30]. In comparison, *crn-6(tm890)* mutant embryos had an average 10.2, 11.0 and 0.7 TUNEL-positive signals, whereas *crn-7(ok866)* mutant embryos had only 6.0, 6.9 and 0.4 TUNEL-positive nuclei, at the comma, 1.5-fold, and 4-fold embryonic stages, respectively. The increased TUNEL-positive signals observed in *crn-6* and *crn-7* mutant embryos were completely abolished by a *ced-3(n2433lf)* mutation (Figure 1B), which blocks almost all cell deaths in *C. elegans* [31], [32], indicating that the TUNEL-positive cells observed in *crn-6* and *crn-7* mutants are apoptotic cells and that CRN-6 and CRN-7 participate in apoptotic DNA degradation but play a lesser role in degrading DNA.

We also examined whether loss of *nuc-1*, *crn-6* or *crn-7* affects the kinetics of apoptosis as in some mutants that are defective in other apoptotic nucleases. We found that the numbers of apoptotic cell corpses in various embryonic stages were similar among N2, *nuc-1(e1392)*, *crn-6(tm890)*, and *crn-7(ok866)* single mutant, and the *crn-7 crn-6*; *nuc-1* triple mutant (Figure 1C), suggesting that loss of all three DNase II genes in *C. elegans* does not affect the activation or progression of cell death and that these three DNase II genes likely act at a late stage of apoptosis.

### CRN-6 plays an auxiliary role to NUC-1 in degrading chromosomal DNA during apoptosis

To investigate whether *crn-6*, *crn-7* and *nuc-1* act redundantly or synergistically to promote apoptotic DNA degradation, we analyzed TUNEL staining in *crn-6; nuc-1* and *crn-7; nuc-1* double mutants and the *crn-7 crn-6; nuc-1* triple mutant (Figure 1A). The *crn-6(tm890); nuc-1(e1392)* double mutant exhibited a modest increase in TUNEL- positive signals in 1.5-fold embryos but contained 58% more TUNEL-positive cells (52 TUNEL signals) in 4-fold embryos than *nuc-1(e1392)* 4-fold embryos (33 TUNEL signals; Figure 1A). In comparison, *crn-6(tm890)* 4-fold embryos had an average of 0.7 TUNEL signal. This observation suggests that CRN-6 plays an auxiliary role to NUC-1 in degrading chromosomal DNA during apoptosis, especially in late embryonic stages. No obvious difference in TUNEL staining was seen between *nuc-1(e1392)* and *crn-7(ok866); nuc-1(e1392)* embryos and between *crn-6(tm890); nuc-1(e1392)* and *crn-7(ok866) crn-6(tm890); nuc-1(e1392)* embryos (Figure 1A), suggesting that CRN-7 plays an insignificant role in apoptotic DNA degradation.

### NUC-1 constitutes the major DNase II activity in *C. elegans*

We next performed an *in vitro* DNA degradation assay to measure acidic DNase II activity in *C. elegans* and to assess the relative contribution of NUC-1, CRN-6 and CRN-7 to this activity [33]. Worm lysate from N2 animals, *nuc-1(e1392), crn-6(tm890)*, and *crn-7(ok866)* single mutants, and their double or triple mutants was prepared and incubated with a supercoiled plasmid DNA in an acidic condition (pH 5.0) (see Materials and Methods). N2, *crn-6(tm890)*, or *crn-7(ok866)* worm lysate displayed a strong endonuclease activity, resulting in complete digestion of the plasmid DNA in 10 minutes at 37°C (Figure 1D, lanes 2, 4, 5). In *nuc-1(e1392)* lysate (Figure 1D, lane 3), the nuclease activity was greatly reduced and the plasmid DNA was mainly in linear (L) and nicked circular (NC) forms, a result of limited cleavage by the residual nuclease activity. In lysate from *crn-6(tm890); nuc-1(e1392)* animals or *crn-7(ok866) crn-6(tm890); nuc-1(e1392)* animals, this residual nuclease activity was lost (Figure 1D, lanes 6 and 7), suggesting that CRN-6 likely provides this residual nuclease activity in the absence of NUC-1 and that CRN-7 contributes a minimal nuclease activity. Altogether, these results indicate that under the acidic pH condition tested (pH 5.0), NUC-1 provides the major DNase II activity and CRN-6 provides the minor DNase II activity in *C. elegans*. This conclusion is consistent with our *in vivo* observation that CRN-6 plays an auxiliary role to NUC-1 in degrading chromosomal DNA during apoptosis.

### The different expression patterns of the three DNase II genes

**Figure 2:**
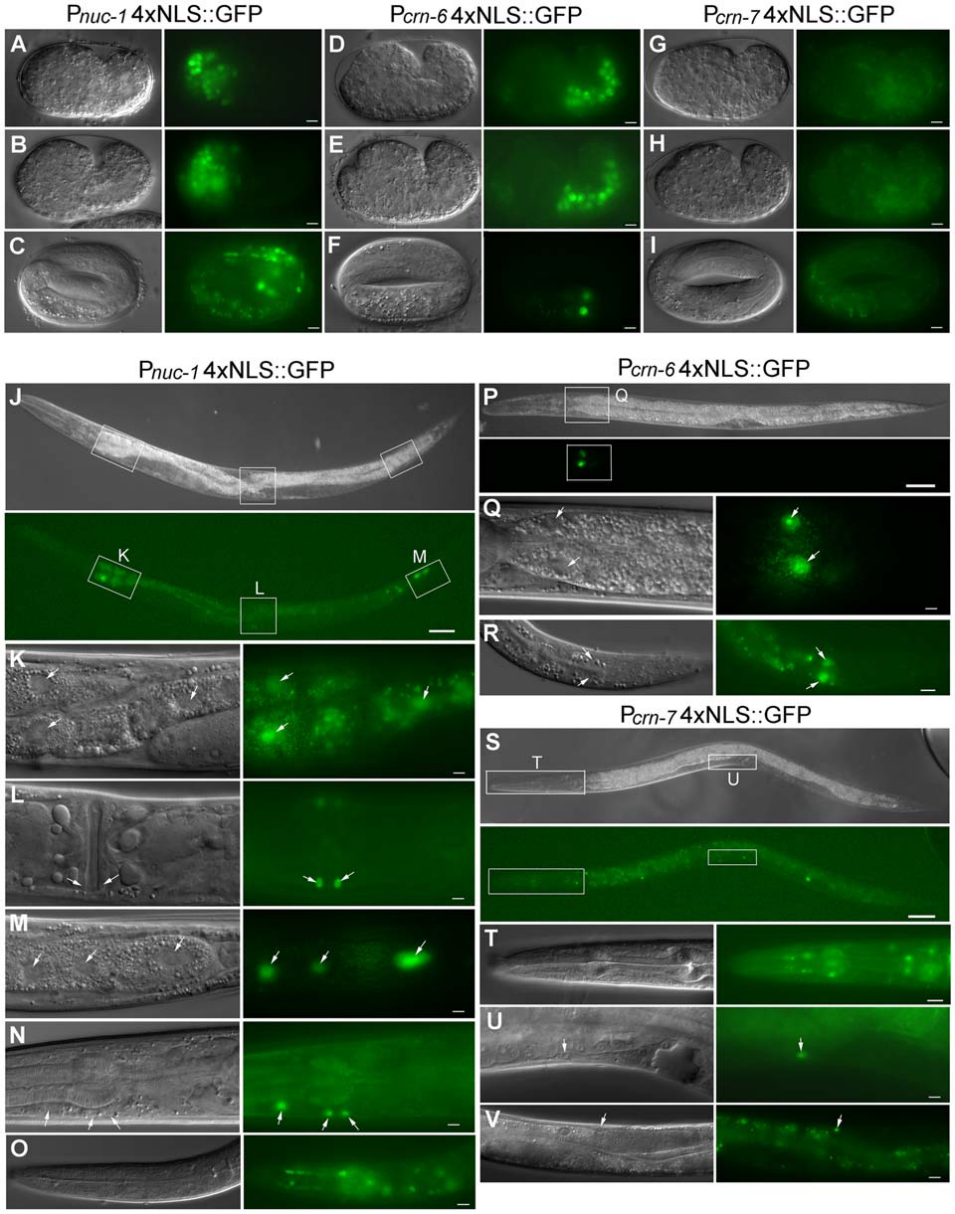
The transcriptional expression patterns of three *C. elegans* DNase II genes. A–I) DIC and GFP images of P*_nuc-1_*4xNLS::GFP, P*_crn-6_*4xNLS::GFP and P*_crn-7_*4xNLS::GFP embryos at comma, 1.5-fold and 4-fold stages (from top to bottom). Scale bars indicate 5 µm. J–V) DIC and GFP images of P*_nuc-1_*4xNLS::GFP, P*_crn-6_*4xNLS::GFP, and P*_crn-7_*4xNLS::GFP larvae. The regions indicated by squares in J are enlarged in K–M. The region indicated by a square in P is enlarged in Q. The regions indicated by squares in S are enlarged in T and U. Arrows in K and Q indicate the anterior intestine cells and arrows in M and R indicate the posterior intestine cells. Arrows in L indicate vulva muscle cells, arrows in N indicate body muscle cells in head, and arrows in U and V indicate body wall muscle cells. The GFP signals in O are from muscle cells and neurons in the head and the GFP signals in T from pharyngeal muscle cells. Scale bars indicate 5 µm except J, P, S (50 µm) and T, V (12.5 µm).

To understand how three DNase II genes might play differential roles in apoptotic DNA degradation, we examined their expression patterns in *C. elegans*. Transcriptional reporter constructs containing the promoter of the DNase II gene fused to GFP with four tandem copies of the nuclear localization signal (NLS) were made and injected into wild-type animals (see Materials and Methods). In P*_nuc-1_*4xNLS::GFP transgenic embryos, GFP was observed exclusively in the head region of comma and 1.5-fold stage embryos. In 3-fold or 4-fold embryos, in addition to the head region, GFP was also observed in anterior intestinal cells (Figure 2A–C). In P*_nuc-1_*4xNLS::GFP transgenic larvae and adults, GFP was strongly expressed in anterior and posterior intestinal cells, and occasionally, in all intestinal cells (data not shown). Weak GFP expression was also detected in the vulva, head and body wall muscle cells (Figure 2J–O). In P*_crn-6_*4xNLS::GFP transgenic embryos, GFP was observed almost exclusively in intestinal precursor cells, but very weak GFP signals were also observed in some posterior cells of early embryos. In late stage P*_crn-6_*4xNLS::GFP embryos, GFP was only observed in a few intestinal cells (Figure 2D–F). In P*_crn-6_*4xNLS::GFP larvae or adults, GFP was consistently detected in intestinal cells, especially in two pairs of the most anterior intestinal cells. Occasionally, GFP was also seen in two pairs of the most posterior intestinal cells (Figure 2P–R). No GFP was detected in P*_crn-7_*4xNLS::GFP transgenic embryos (Figure 2G–I), however, GFP signals were observed in the head muscle and body wall muscle cells in P*_crn-7_*4xNLS::GFP larvae (Figure 2S–V). Therefore, except for a few anterior intestinal cells in 4-fold embryos, the embryonic expression patterns of the three DNase II genes in *C. elegans* do not seem to overlap. In larvae and adults, however, both *nuc-1* and *crn-6* are expressed in some intestinal cells and there is partial overlap of *nuc-1* and *crn-7* expression in the head and body wall muscle cells.

### NUC-1 and CRN-6 affect apoptotic DNA degradation in many cells despite restricted expression patterns

**Figure 3:**
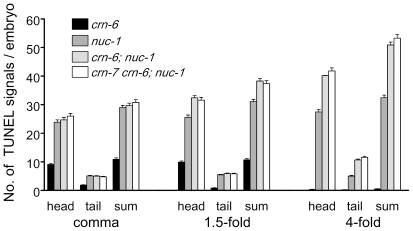
TUNEL signals at the head and tail regions of embryos deficient in three DNase II genes. An arbitrary line at the bottom of the pharynx was used to separate the embryo into two regions, head and tail. TUNEL signals were scored in both regions in different mutants. The combined TUNEL signals from both head and tail regions (sum) were also indicated. *crn-6(tm890)*, *crn-7(ok866)*, and *nuc-1(e1392)* were the alleles used. At least 24 embryos from each stage were scored. Error bars indicate SEM.

Since *nuc-1* and *crn-6* are expressed in different regions of embryos (anterior and posterior embryos, respectively), we examined whether they mediate apoptotic DNA degradation in the regions where they are expressed, by scoring TUNEL signals in the head and tail regions of *nuc-1(e1392), crn-6(tm890)*, and *crn-6; nuc-1* double mutant embryos. In both *nuc-1(e1392)* and *crn-6(tm890)* embryos, the majority of TUNEL signals were seen in the head region and some were seen in the tail region (Figure 3). Similarly, in 1.5-fold and 4-fold *crn-6(tm890); nuc-1(e1392)* embryos, the increased TUNEL signals caused by *crn-6(tm890)* over those of the *nuc-1(e1392)* mutant were seen mostly in the head region and some in the tail region. These results indicate that *nuc-1* and *crn-6* affect apoptotic DNA degradation in both anterior and posterior regions of embryos, despite their restricted expression patterns in the anterior and the posterior regions of the embryo, respectively. This finding is also consistent with the observation that most *C. elegans* embryonic cell deaths occur in the anterior region of the embryo [30] and thus most TUNEL-positive signals are seen in the head region of the *nuc-1* and *crn-6* mutant embryos. The fact that both NUC-1 and CRN-6 affect apoptotic cells outside of their expression areas suggests that these two nucleases may be secreted (as predicted from their sequences; Figure S1A) and diffuse to other regions of the embryo to affect apoptotic DNA degradation.

### NUC-1, but not CRN-6 or CRN-7, mediates degradation of chromosome DNA in ventral cord apoptotic cells and bacterial DNA in intestine

In addition to causing accumulation of TUNEL-positive signals in embryos, loss of *nuc-1* results in abnormally persistent DNA in the ventral cord and undigested bacterial DNA in the lumen of intestine [26], both of which can be stained by a vital DNA dye, Syto 11 [23] (see Materials and Methods). For example, *nuc-1(e1392)* L2 larvae treated with Syto 11 showed strong staining of intestine and multiple apoptotic cells in the ventral nerve cord (Figure S2A), which is not seen in wild-type animals treated with Syto 11 (Figure S2B). The ventral cord nuclei strongly stained by Syto 11 are known as “pycnotic nuclei” because of their highly condensed DNA content [26], [31] and derive from cells that undergo apoptosis in the W and P1-P12 neuroblast lineages in hermaphrodites [34]. Seven of these pycnotic nuclei posterior to the vulva are from P9-P12 lineages and were scored for Syto 11 staining in this study. *nuc-1(e1392)* late L1 to early L3 larvae had an average 6.5 pycnotic nuclei and 89% of L3-L4 larvae had strong gut staining (Table 1). In contrast, wild type, *crn-6(tm890)*, *crn-7(ok866)*, or *crn-7(ok866) crn-6(tm890)* larvae did not contain pycnotic nuclei in the ventral cord and few of them had strong gut staining when stained with Syto 11 (Table 1). These results suggest that NUC-1 is the major nuclease, if not the only nuclease, involved in degrading DNA of apoptotic cells in the ventral cord and DNA of ingested bacteria. Consistent with this conclusion, we did not observe any further increase in pycnotic nuclei or increased number of animals with strong gut staining in *crn-6(tm890) crn-7(ok866)*, *crn-6(tm890); nuc-1(e1392), crn-7(ok866); nuc-1(e1392)*, or *crn-7(ok866) crn-6(tm890); nuc-1(e1392)* animals (Table 1). Nor did we observe any pycnotic nuclei or increased number of animals with strong gut staining in *crn-6(tm890); nuc-1(e1392)/+* or *crn-7(ok866) crn-6(tm890); nuc-1(e1392)/+* animals (Table 1), in which the *nuc-1(e1392)/+* heterozygosity could provide a sensitized background for revealing the potential contribution of *crn-6* and *crn-7* to these two DNA degradation events. Therefore, NUC-1 appears to be the only DNase II nuclease that has a function in degrading DNA in post-embryonic apoptotic cells and bacterial DNA in intestine.

**Table 1:** *nuc-*1, but not *crn-6* or *crn-7*, mediates degradation of DNA in ventral cord apoptotic cells and DNA of ingested bacteria.

Genotype	No. of pycnotic nuclei^a^ (n)	Strong gut staining^b^ (n)
N2	0.1±0.7 (30)	4% (120)
*nuc-1(e1392)*	6.5±0.7 (75)	89% (115)
*crn-6(tm890)*	0.0±0.0 (30)	7% (105)
*crn-7(ok866)*	0.0±0.0 (60)	3% (60)
*crn-7(ok866) crn-6(tm890)*	0.04±0.3 (72)	7% (57)
*crn-6(tm890); nuc-1(e1392)*	6.3±0.7 (56)	86% (107)
*crn-7(ok866); nuc-1(e1392)*	6.2±0.8 (64)	83% (64)
*crn-7(ok866) crn-6(tm890); nuc-1(e1392)*	6.3±0.8 (72)	85% (62)
*nuc-1(e1392)/+* c	0.0±0.0 (42)	2% (46)
*crn-6(tm890); nuc-1(e1392)/+* c	0.0±0.0 (41)	2% (46)
*crn-7(ok866) crn-6(tm890); nuc-1(e1392)/+* c	0.0±0.0 (42)	0% (38)

Syto 11 staining was carried out as described in Materials and Methods.

a Mixed stage larval animals (late L1 to early L3) were scored for the number of ventral cord pycnotic nuclei posterior to the vulva. “n” indicates the number of animals scored. The data shown are mean±SD (standard deviation).

b Strong gut staining that represents undigested bacterial DNA in intestine was scored at L3–L4 larval stages. “n” indicates the number of animals scored.

c *nuc-1/+* heterozygote is actually *nuc-1*/*axIs36*. See Materials and Methods for details.

### CRN-6, but not CRN-7, can partially substitute for NUC-1 in mediating apoptotic DNA degradation

We next tested whether three *C. elegans* DNase II homologues can functionally substitute for one another. We generated transgenes that drive the expression of NUC-1::GFP, CRN-6::GFP, or CRN-7::GFP under the control of the *nuc-1* promoter (P*_nuc-1_*) and then examined whether these fusion proteins can rescue the *nuc-1(e1392)* defects (Table S1 and Materials and Methods). To avoid overexpressing these fusion proteins in *C. elegans*, we used the ballistic bombardment technique to integrate these expression constructs into *C. elegans* genome, generating low-copy integrated lines with varying GFP expression intensities (Table S1).

**Figure 4:**
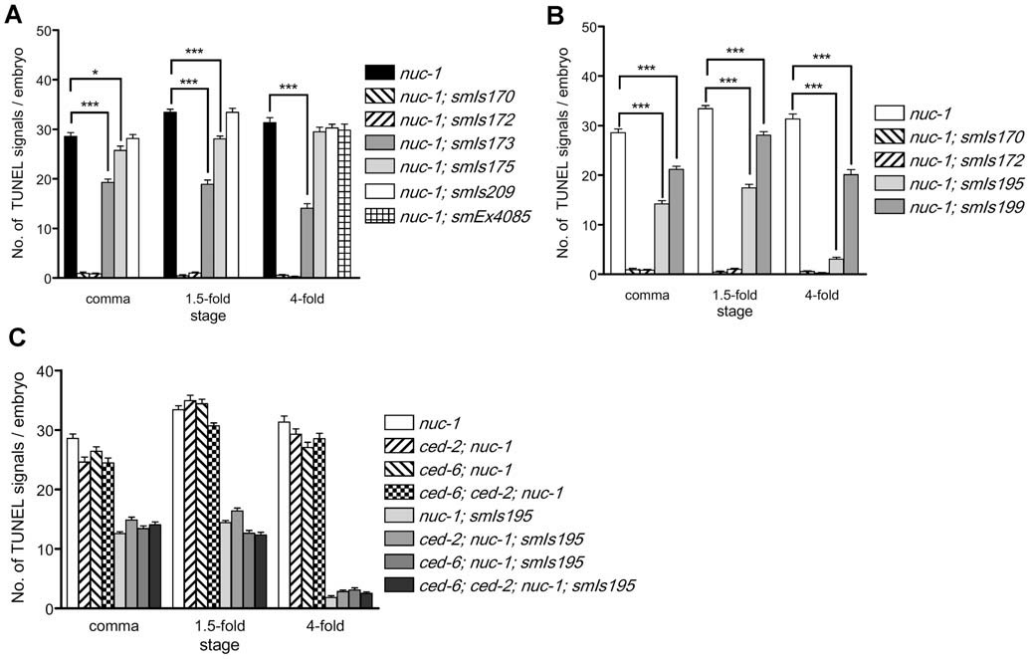
TUNEL assays in embryos expressing three DNase II genes under the control of different promoters. A) TUNEL staining of transgenic *nuc-1(e1392)* embryos carrying different integrated transgenes that express three DNase II genes under the control of the *nuc-1* promoter. *smIs170* and *smIs172* are P*_nuc-1_nuc-1::gfp* transgenic lines. *smIs173* and *smIs175* are P*_nuc-1_crn-6::gfp* transgenic lines. *smIs209* is the P*_nuc-1_crn-7::gfp* transgenic line. *smEx4085* is an extrachromosomal transgenic line containing P*_nuc-1_crn-7::gfp* and P*_myo-2_gfp* as a transgenic marker. *smEx4085* transgenic embryos were doubly stained with TUNEL and anti-GFP antibody (to identify transgenic embryos). Only 4-fold embryos were scored, since GFP expression derived from P*_myo-2_gfp* was not observed until late embryonic stages. B) TUNEL staining of transgenic *nuc-1(e1392)* embryos carrying different integrated transgenes that express *nuc-1::gfp* under the control of the *nuc-1* or the *crn-6* promoter. *smIs195* and *smIs199* are P*_crn-6_nuc-1::gfp* transgenic lines. C) Loss of the cell corpse engulfment gene *ced-2* or *ced-6* or both does not affect the rescue of the *nuc-1(e1392)* TUNEL defect by *smIs195*. *ced-2(e1752)* and *ced-6(n2095)* alleles were used to construct various strains. In all experiments, at least 25 embryos at each embryonic stage were scored. Error bars indicate SEM. In A and B, the significance of differences in two different genetic backgrounds was determined by student *t* test. * *P*<0.05, *** *P*<0.001.

In two P*_nuc-1_nuc-1::gfp* lines (*smIs170* and *smIs172*), no GFP was detected in embryos but weak GFP signals could be observed in the two anterior intestinal cells in larvae (Table S1). Both transgenes completely rescued the TUNEL defect of *nuc-1(e1392)* embryos (Figure 4A), indicating that the *nuc-1* promoter used in the transgenes is sufficient to drive *nuc-1::gfp* expression to rescue the TUNEL defect of the *nuc-1(e1392)* mutant. In two P*_nuc-1_crn-6::gfp* lines (*smIs173* and *smIs175*), CRN-6::GFP was seen in the head region of embryos (Figure S3A–D). In transgenic larvae, GFP was detected in the anterior and posterior intestine cells, vulva muscle, many cells in the head region, and intestinal lumen (Figure S3E–H). The GFP expression patterns of P*_nuc-1_crn-6::gfp* transgenes are similar to those seen in P*_nuc-1_*4xNLS::GFP animals (Figure 2A–C, J–O), except that CRN-6::GFP was excluded from nuclei in P*_nuc-1_crn-6::gfp* transgenic animals. *smIs173* (with 31–32 copies of the construct) exhibited significant rescue of the TUNEL defect of the *nuc-1(e1392)* mutant in all three embryonic stages, whereas *smIs175* (with 7–8 copies) showed mild rescue in comma and 1.5-fold *nuc-1(e1392)* embryos and no rescue in 4-fold *nuc-1(e1392)* embryos (Figure 4A). These results indicate that CRN-6 can partially substitute for NUC-1 in resolving 3′ OH DNA breaks generated in apoptotic cells when expressed from the *nuc-1* promoter. However, the nuclease activity of CRN-6 appears to be much weaker than that of NUC-1, as suggested by the nuclease activity assay (Figure 1D). As a result, the P*_nuc-1_crn-6::gfp* transgene (*smIs173*) with a higher copy number (31–32 copies) and stronger GFP fusion expression than either P*_nuc-1_nuc-1::gfp* transgenes (*smIs170* and *smIs172)* only achieved partial rescue of the *nuc-1* mutant. In three P*_nuc-1_crn-7*::*gfp* transgenic lines, *smIs209–211* (with 7–8, 3–4 and 1–2 copies, respectively), we did not detect obvious GFP signals in embryos or in larvae (Table S1). All three transgenes failed to rescue the *nuc-1* TUNEL defect (Figure 4A; data not shown). In animals carrying a high copy number extrachromosomal array (*smEx4085*) that contains P*_nuc-1_crn-7*::*gfp*, although we observed bright CRN-7::GFP signals in the anterior region of the transgenic embryos, we failed to detect rescue of the *nuc-1* TUNEL defect (Figure 4A and Table S1). These results indicate that CRN-7 cannot substitute for NUC-1 to mediate apoptotic DNA degradation.

In transgenic larvae carrying the P*_nuc-1_nuc-1*::*gfp* transgenes, *smIs170* and *smIs172*, NUC-1::GFP completely rescued the pycnotic nuclei phenotype of the *nuc-1* mutant (Table 2). No pycnotic nuclei were seen in *nuc-1(e1392); smIs170* or *nuc-1(e1392); smIs172* larvae, whereas an average of 6.7, 6.3, 6.4, and 5.5 pycnotic nuclei posterior to the vulva were observed in different larval stages of the *nuc-1(e1392)* mutant (late L1, L2, early L3, and L3–L4). Partial rescue of the “pycnotic nuclei” phenotype was observed in *nuc-1(e1392)* animals carrying a higher copy number of the P*_nuc-1_crn-6*::*gfp* transgene (*smIs173*), especially in the late larval stages (Table 2). Only 1.0 pycnotic nucleus was seen in L3-L4 stage, compared with 5.5 pycnotic nuclei observed in *nuc-1(e1392)* animals at the same stage. Poor or no rescue of the “pycnotic nuclei” phenotype was observed in *nuc-1(e1392)* animals with a lower copy number of the P*_nuc-1_crn-6*::*gfp* transgene (*smIs175*) or with four different P*_nuc-1_crn-7*::*gfp* transgenes (*smIs209-211, smEx4085*; Table 2). Taken together, these results suggest that CRN-6, but not CRN-7, can partially substitute for NUC-1 in mediating embryonic and post-embryonic apoptotic DNA degradation.

**Table 2:** CRN-6 can partially substitute for NUC-1 in mediating degradation of DNA in ventral cord apoptotic cells.

Genotype^a^	N. P. C. in late L1^b^	N. P. C. in L2^b^	N. P. C. in early L3^b^	N. P. C. in L3–4^b^	Strong gut staining^c^ (n)
*nuc-1*	6.7±0.6 (27)	6.3±0.8 (22)	6.4±0.6 (26)	5.5±1.2 (22)	89% (115)
*smIs170*	0.5±1.0 (24)	0.0±0.0 (22)	0.0±0.0 (21)	0.0±0.0 (24)	14% (43)
*smIs172*	0.0±0.0 (24)	0.0±0.0 (26)	0.0±0.0 (24)	0.0±0.0 (24)	5% (44)
*smIs173*	5.0±1.4 (22)	2.5±1.2 (34)	1.3±1.4 (38)	1.0±1.1 (58)	76% (67)
*smIs175*	6.1±1.0 (29)	5.8±0.8 (29)	5.4±1.0 (32)	4.4±0.9 (35)	78% (83)
*smIs209*	5.8±1.9 (4)	5.8±0.9 (24)	5.9±1.0 (16)	4.6±1.0 (37)	80% (64)
*smIs210*	6.7±0.5 (9)	6.3±0.8 (27)	6.0±0.9 (6)	N. D.	78% (46)
*smIs211*	6.5±0.5 (10)	5.9±1.1 (23)	5.0±1.8 (8)	N. D.	91% (23)
*smIs195*	5.9±0.8 (16)	4.5±1.2 (18)	2.6±1.4 (18)	1.6±1.3 (43)	13% (53)
*smIs199*	6.3±0.9 (26)	6.1±0.9 (29)	5.6±0.8 (28)	5.0±1.7 (26)	88% (52)
*smEx4085* d	6.3±0.6 (11)	6.3±0.7 (23)	6.5±0.5 (13)	N.D.	88% (42)

a All strains except *nuc-1(e1392)* were in *unc-119(ed3)*; *nuc-1(e1392)* background. *smIs170* and *smIs172* are P*_nuc-1_nuc-1::gfp* transgenic lines. *smIs173* and *smIs175* are P*_nuc-1_crn-6::gfp* transgenic lines. *smIs209*-*smIs211* are P*_nuc-1_crn-7::gfp* transgenic lines. *smIs195* and *smIs199* are P*_crn-6_nuc-1::gfp* transgenic lines (see Table S1 for details)

b N. P. C. indicates the number of ventral cord pycnotic nuclei, which were scored at different larval stages. “n” indicates the number of animals scored. The data shown are mean±SD.

c All gut staining was scored at L3–L4 stages. “n” indicates the number of animals scored.

d *smEx4085* is an extrachromosomal transgene array containing P*_nuc-1_crn-7::gfp* and P*_myo-2_gfp.*

### Neither CRN-6 nor CRN-7 can substitute for NUC-1 in digesting bacterial DNA in intestine

We also tested whether the above transgenes can restore degradation of bacterial DNA in the intestine of *nuc-1(e1392)* animals. Two P*_nuc-1_nuc-1*::*gfp* transgenes, *smIs170* and *smIs172*, can strongly rescue the defect in digesting bacterial DNA (Table 2), whereas none of the P*_nuc-1_crn-6*::*gfp* and P*_nuc-1_crn-7*::*gfp* transgenes displayed obvious rescuing activity, despite of the secretion of CRN-6::GFP into the intestine (Figure S3H). These results suggest that CRN-6 and CRN-7 cannot substitute for NUC-1 in digesting bacterial DNA in intestine and that only NUC-1 has this activity.

Interestingly, when NUC-1::GFP was expressed under the control of the *crn-6* gene promoter (P*_crn-6_nuc-1*::*gfp*), one transgene (*smIs195*, 5–6 copies) showed strong rescue of the gut staining in the *nuc-1(e1392)* mutant (Table 2). However, a second transgene with a lower copy number (*smIs199*, 2–3 copies) failed to do so. Therefore, the *crn-6* promoter is capable of directing the expression of NUC-1 to mediate degradation of bacterial DNA in intestine but the level of NUC-1 expression appears to be critical for this function.

### *nuc-1* can still promote apoptotic DNA degradation in both embryos and larvae when its expression is restricted to intestine cells

Since the expression patterns of *crn-6* and *nuc-1* do not overlap in embryos (except a few cells at the 4-fold stage embryos) and CRN-6 is expressed specifically in the posterior half of embryos (Figure 2A–F), we examined how NUC-1 expressed under the control of the *crn-6* promoter (P*_crn-6_nuc-1*::*gfp*) affects DNA degradation in apoptotic cells, which occur mostly in the anterior half of the embryos. In animals carrying two P*_crn-6_nuc-1*::*gfp* transgenes (*smIs195* and *smIs199*), no obvious GFP signal was observed in embryos and only weak GFP signals could be seen in the two anterior intestinal cells. In contrast, in animals carrying two P*_crn-6_crn-6::gfp* transgenes (*smIs187* and *smIs189*) that fully rescued the TUNEL defect of *crn-6(tm890)* embryos (Figure S4A), strong GFP signals were detected exclusively in cytoplasm of intestinal cells throughout the embryonic stages and in two pairs of anterior intestinal cells in larvae and adults (Figure S4B–F; data not shown). This CRN-6::GFP expression pattern is similar to the GFP expression pattern in P*_crn-6_*4xNLS-GFP transgenic animals (Figure 2D–F, P–R). The significantly stronger GFP intensity observed in P*_crn-6_crn-6::gfp* transgenic lines than in P*_crn-6_nuc-1*::*gfp* lines and stronger GFP intensity observed in P*_nuc-1_crn-6::gfp* transgenic lines than in P*_nuc-1_nuc-1*::*gfp* lines (Table S1) indicate that NUC-1::GFP is either poorly expressed or unstable in worm embryos. Despite of the very low expression level of NUC-1::GFP, *smIs195* (5–6 copies of P*_crn-6_nuc-1*::*gfp*) still resulted in 50% reduction of the TUNEL signals in *nuc-1(e1392)* comma and 1.5-fold embryos and almost completely rescued the TUNEL defect of *nuc-1(e1392)* 4-fold embryos (Figure 4B). Even in the line with a lower copy number of P*_crn-6_nuc-1*::*gfp* (*smIs199*, 2–3 copies), partial rescue of the TUNEL defect of *nuc-1(e1392)* embryos was detected (Figure 4B). These results indicate that NUC-1 can still function to resolve 3′ OH DNA breaks in apoptotic cells of the whole embryo, even though its expression is restricted to intestine cells at the posterior region of the embryo under the *crn-6* promoter. These observations further suggest that NUC-1 is secreted and then taken up by cells in the whole embryo to achieve its widespread DNA degradation activity.

Similar to what was observed in the embryo, *smIs195* partially rescued the pycnotic nuclei phenotype in *nuc-1(e1392)* larvae, especially in later larval stages (Table 2). For example, an average of 1.6 pycnotic nuclei were seen in *smIs195; nuc-1(e1392)* L3–L4 larvae, compared with 5.5 pycnotic nuclei seen in *nuc-1(e1392)* larvae at the same stage (Table 2). In contrast, a lower copy number of P*_crn-6_nuc-1*::*gfp* transgene (*smIs199*, 2–3 copies) failed to rescue the pycnotic nuclei phenotype of *nuc-1(e1392)* larvae.

### NUC-1 expressed in the head can mediate apoptotic DNA degradation in the posterior ventral cord

**Figure 5:**
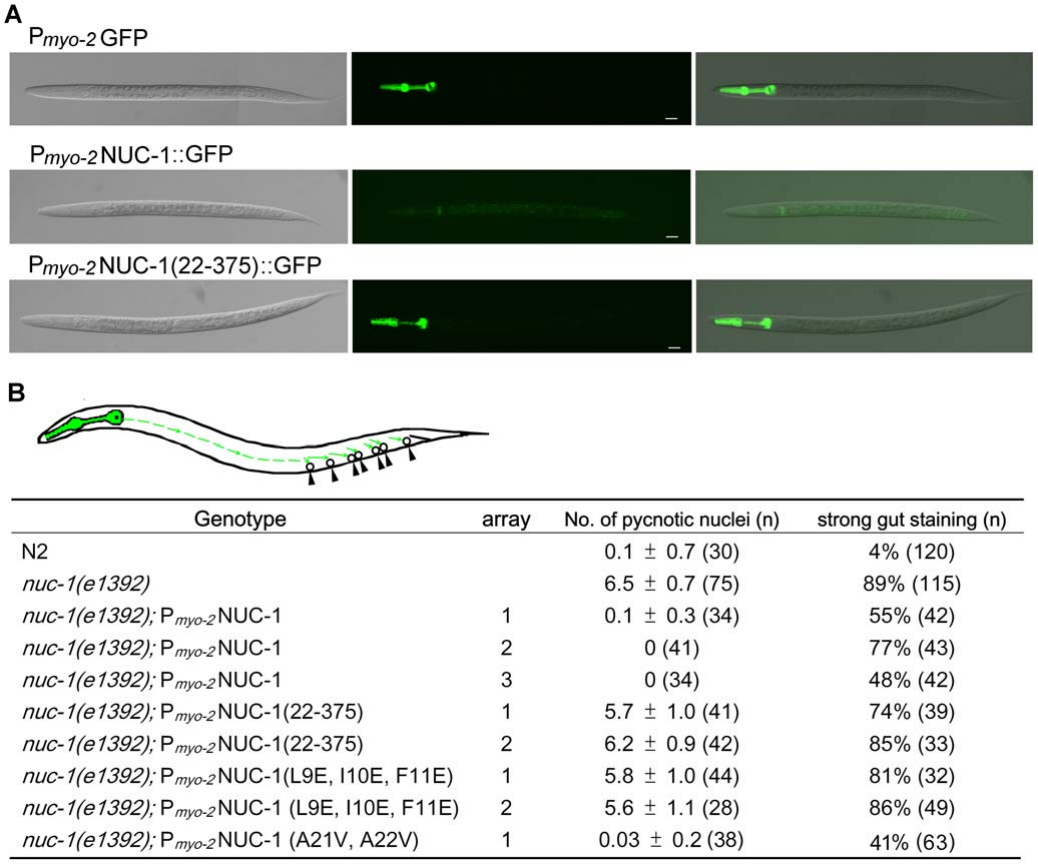
NUC-1 expressed in and secreted from the head can rescue the Nuc-1 defect in the tail cells. A) Removal of the NUC-1 signal peptide prevents NUC-1 from being secreted and transported out of the pharynx. DIC, GFP, and merge images of L2 larvae from P*_myo-2_*GFP, P*_myo-2_*NUC-1::GFP, and P*_myo-2_*NUC-1(22–375)::GFP transgenic animals are shown. In P*_myo-2_*NUC-1(22–375)::GFP, the first 21 amino acids of NUC-1, the predicted signal peptide, is deleted. The exposure time of the GFP image is 4 ms for P*_myo-2_*GFP, 160 ms for P*_myo-2_*NUC-1::GFP, and 40 ms for P*_myo-2_*NUC-1(22–375)::GFP, respectively. Scale bars indicate 12.5 µm. B) Secretion of NUC-1 expressed in the pharynx is important for rescuing DNA degradation defect in apoptotic cells of the posterior ventral cord. The apoptotic cells scored in the posterior ventral cord are indicated with circles and arrowheads. NUC-1 or various NUC-1 mutants is expressed specifically in the pharynx (shown in green) under the control of the *myo-2* gene promoter. Some of the proteins [NUC-1 or NUC-1(A21V, A22V)] are likely secreted and transported out of the pharynx to the posterior end of the animals (green dash lines), where they are retaken up by the apoptotic cells indicated to mediate DNA degradation. In NUC-1 (L9E, I10E, F11E), three key hydrophobic amino acids in the signal peptide are replaced by charged residues, Glutamates. Both NUC-1(22–375) and NUC-1(L9E, I10E, F11E) are predicted to be non-secretory by the SignalP 3.0 program (see Figure S1 for details). In NUC-1 (A21V, A22V), two Alanine residues are replaced by Valines but the protein is predicted to remain secretory by the SignalP 3.0 program. The number of pycnotic nuclei was scored in the posterior ventral cord of mixed stage larvae (late L1 to early L3). Gut staining was scored at L3–L4 stages. “n” indicates the number of animals scored. The data shown are mean±SD.

To assess how NUC-1 affects DNA degradation in apoptotic cells where NUC-1 is not expressed, we expressed NUC-1 under the control of the *myo-2* gene promoter, which drives gene expression exclusively in the pharyngeal muscle in the head (upper panel, Figure 5A)[35]. Remarkably, in animals carrying P*_myo-2_*NUC-1, all three transgenic lines completely rescued the pycnotic nuclei phenotype of the *nuc-1(e1392)* mutant and partially rescued the defect in degrading intestinal bacterial DNA (Figure 5B). This result suggests that NUC-1 synthesized in the pharynx must be secreted, travel more than half the distance of the animal's body length, and be retaken up by cells in the posterior ventral cord to mediate DNA degradation (see cartoon in Figure 5B). Indeed, we barely detected any NUC-1::GFP in the pharynx of animals carrying P*_myo-2_*NUC-1::GFP (middle panel, Figure 5A) but observed strong NUC-1::GFP in the pharynx of animals carrying P*_myo-2_*NUC-1(22–375)::GFP (bottom panel, Figure 5A), in which the NUC-1 signal peptide (amino acids 1–21) was deleted and the mutant NUC-1 protein was not secreted. Importantly, P*_myo-2_*NUC-1(22–375) failed to rescue the pycnotic nuclei phenotype and the defect in degrading intestinal bacterial DNA in the *nuc-1(e1392)* mutant (Figure 5B), indicating that secretion of NUC-1 is critical for its activity in mediating degradation of DNA in apoptotic cells of the posterior ventral cord and in intestinal lumen. Similarly, expression of another non-secretory NUC-1 mutant, NUC-1(L9E, I10E, F11E), did not rescue of the DNA degradation defects of the *nuc-1(e1392)* mutant (Figure 5B). On the other hand, expression of a NUC-1 mutant, NUC-1(A21V, A22V), which is predicted to remain secretory, fully rescued the pycnotic nuclei phenotype of the *nuc-1(e1392)* mutant (Figure 5B). Altogether, these results indicate that NUC-1 is secreted and transported to, or diffuses to distant regions, where it is retaken up by cells to mediate DNA degradation.

### NUC-1 can act in apoptotic cells to resolve 3′ OH DNA breaks

In mammals, DNase II is thought to act in engulfing cells, whereas in *C. elegans* NUC-1 is implicated to function in apoptotic cells [9], [23]. To address this critical issue, we take advantage of the observation that *nuc-1* expressed in posterior intestine cells under the control of the *crn-6* promoter can still promote apoptotic DNA degradation in the anterior region of the embryo (Figure 4B). This finding indicates that NUC-1 must be secreted and retaken up by cells in the anterior region of the embryo to mediate DNA degradation. We then tested whether blocking cell corpse engulfment affects NUC-1-mediated DNA degradation. As shown in Figure 4C, NUC-1 expressed from the *smIs195* transgene (P*_crn-6_nuc-1*::*gfp*) still rescued the TUNEL defect in *ced-2(e1752); nuc-1(e1392)* or *ced-6(n2095); nuc-1(e1392)* embryos, in which cell corpse engulfment is severely compromised, or the TUNEL defect of the *ced-6(n2095); ced-2(e1752); nuc-1(e1392)* triple mutant, in which both engulfment pathways are blocked and few apoptotic cells are engulfed by phagocytes [36]. These results indicate that the *nuc-1* rescuing activity is not contributed by neighboring engulfing cells. Instead, NUC-1 can act in apoptotic cells to digest 3′OH DNA breaks.

## Discussion

DNase II enzymes are the major acidic nucleases in cells and have been shown to play important roles in apoptosis and animal development [15]. In both mammals and *C. elegans*, multiple DNase II nucleases exist but their relative contributions to apoptosis and animal development remain unclear. Moreover, how DNase II enzymes act to promote apoptotic DNA degradation remain controversial. In this study, we carried out comprehensive analysis of *nuc-1*, *crn-6*, and *crn-7*, three genes that encode *C. elegans* DNase II enzymes. Our results indicate that three *C. elegans* DNase II homologues play differential roles in both apoptotic and non-apoptotic DNA degradation. Strikingly, we discover that NUC-1 is a secreted nuclease that can travel a long distance and be retaken up at distant sites to mediate DNA degradation in apoptotic cells.

### NUC-1, CRN-6 and CRN-7 do not affect activation or timing of apoptosis but play differential roles in mediating both apoptotic and non-apoptotic DNA degradation

The identification of multiple DNase II homologues in *C. elegans* and mammals has raised two important questions regarding how they might act to promote DNA degradation [15]. 1) Are they expressed in different cells or tissues? and 2) Do they play different or redundant roles in DNA degradation? To address these questions, we analyzed the expression patterns of transcriptional reporters that express 4xNLS::GFP under the control of the three *C. elegans* DNase II gene promoters. We found that the embryonic expression patterns of the three DNase II genes in *C. elegans* do not overlap, except for a few anterior intestinal cells in 4-fold embryos. In larvae and adults, both *nuc-1* and *crn-6* are expressed in some intestinal cells and there is partial overlap in *nuc-1* and *crn-7* expression in the head and body wall muscle cells (Figure 2). The largely non-overlapping expression patterns of *C. elegans* DNase II genes differ from those of human DNase II enzymes, which either is ubiquitously expressed (DNase IIα) or has restricted expression in a few cell types (DNase IIβ) [17]–[19]. Such expression patterns also raise the interesting question of whether they play different roles in *C. elegans*.

In TUNEL assays that detect 3′ OH DNA breaks generated during apoptosis, loss of either *nuc-1, crn-6*, or *crn-7* causes increased number of TUNEL-positive cells in mutant embryos (Figure 1A and 1B), suggesting that all three genes contribute to resolving 3′ OH DNA breaks generated in apoptotic cells. Moreover, NUC-1 and CRN-6 appear to act synergistically to promote apoptotic DNA degradation, as increased numbers of TUNEL-positive cells are seen in the *crn-6*; *nuc-1* double mutant, especially in the late embryonic stages (Figure 1A). However, like *nuc-1, crn-6*, or *crn-7* single mutants, loss of all three DNase II genes in the *crn-7 crn-6; nuc-1* triple mutant fails to affect either the timing of apoptosis or the activation of apoptosis (Figure 1C). This observation rules out the possibility that genetic redundancy masks a role of DNase II genes in cell death activation or in regulating the kinetics of apoptosis and is consistent with the model that DNase II genes act at a later stage of apoptosis after cell death activation and execution have occurred [3]. This is also the first genetic analysis of defects caused by loss of all DNase II genes in animals.

The TUNEL analysis of the *nuc-1, crn-6* or *crn-7* single mutants as well as their double or triple mutants also reveals that three DNase II genes contribute differentially to the apoptotic DNA degradation process, with NUC-1 playing a major role, CRN-6 playing an auxiliary role, and CRN-7 playing a negligible role in apoptotic DNA degradation during embryo development (Figure 1A). The results of Syto 11 DNA dye staining of *C. elegans* larvae suggest that NUC-1, but not CRN-6 or CRN-7, is solely responsible for degrading chromosomal DNA in ventral cord apoptotic cells and for degrading DNA of ingested bacteria in the intestine (Table 1). These results point to a dominant role of NUC-1 in all three facets of DNA degradation mediated by acidic nucleases in *C. elegans* and are consistent with our nuclease activity assays (Figure 1D) and our promoter swapping experiments (Figure 4A and Table 2), which clearly indicate that NUC-1 provides the major acidic nuclease activity in *C. elegans* and CRN-6 contributes minor acidic nuclease activity.

Although we did not observe an obvious role for CRN-7 in any of the DNA degradation events, loss of *crn-7* does cause a significant reduction in animal brood size when combined with *crn-6* and *nuc-1 lf* mutations. For example, the brood size of the *crn-6(tm890); nuc-1(e1392)* double mutant is approximately 87% of that in wild type animals or 75–85% of that in individual *crn-6*, *nuc-1*, or *crn-7* single mutants (Table S2). In contrast, the brood size of the *crn-7 crn-6; nuc-1* triple mutant is less than 50% of that in wild type animals or *crn-6*, *nuc-1* or *crn-7* single mutants. The brood size reduction observed in the *crn-7 crn-6; nuc-1* triple mutant is not suppressed by a strong loss-of-function mutation in *ced-3 (n2433)*, suggesting that this defect is not related to apoptosis and that the three DNase II enzymes might have a redundant but important role in germ line development.

### CRN-6 but not CRN-7 can partially substitute for NUC-1 in mediating apoptotic DNA degradation

In an effort to assess the functional interchangeability of three *C. elegans* DNase II genes, we expressed them under the control of the *nuc-1* promoter and examined the ability of each transgene in rescuing the *nuc-1(e1392)* defects. Despite of an overall sequence similarity between CRN-6 and NUC-1, especially at the catalytic site (Figure S1A), and despite of stronger CRN-6::GFP expression than NUC-1::GFP expression under the control of the same *nuc-1* promoter (Figure S3), CRN-6 only partially rescues the TUNEL defect and the pycnotic nuclei phenotype of the *nuc-1(e1392)* mutant and completely fails to rescue the defect in degrading bacterial DNA in intestine (Figure 4A and Table 2), whereas NUC-1 completely rescues all *nuc-1* defects. In addition, CRN-7 fails to rescue any of the *nuc-1* defects when expressed under the control of the *nuc-1* promoter. Since all three DNase II genes were expressed under the same *nuc-1* promoter and hence were expressed in the same cells with the same pH environment, the drastically different *in vivo* rescuing activities most likely reflect different intrinsic nuclease activities of the three DNase II enzymes and correlate well with their acid nuclease activities revealed by the *in vitro* nuclease activity assays.

### NUC-1 is a secreted nuclease that can travel a long distance and be retaken up at distant sites to mediate DNA degradation in apoptotic cells

It has been suggested that the acting site of DNase II in mammals and *Drosophila* is in phagocytes where DNA of internalized apoptotic cells or bacteria is degraded by DNase II localized in lysosomes [10], [37], [38]. On the other hand, genetic analysis in *C. elegans* suggests that blocking cell corpse engulfment by engulfment defective mutations such as *ced-2(lf)* or *ced-6(lf)* mutations clearly does not affect apoptotic DNA degradation mediated by *nuc-1* [23], arguing that NUC-1 may act in apoptotic cells to promote DNA degradation. Interestingly, almost all DNase II genes are predicted to encode proteins with a signal peptide [15], [39], and some, such as the human DNase II, has been shown to be secreted into the media when expressed in cultured cells [40]. However, it is unclear why DNase II enzymes are secreted, whether they are indeed secreted *in vivo*, and how secretion of DNase II might affect its *in vivo* DNA degradation functions. In our analysis of *crn-6* and *nuc-1* expression patterns through transcriptional reporters, *crn-6* transcription is found to occur specifically in the posterior half of the embryo, whereas *nuc-1* is transcribed specifically in the anterior half of the embryo in a largely non-overlapping fashion (Figure 2). Yet both genes affect apoptotic DNA degradation in both the head and the tail regions of the embryo (Figure 3), suggesting that NUC-1 and CRN-6 can cross their expression borders to affect apoptotic cells in other regions, which is a typical feature of secreted proteins. Similarly, in one of our promoter swapping experiments (P*_crn-6_nuc-1*::*gfp*), *nuc-1* was expressed in the posterior half of embryos under the control of the *crn-6* promoter, yet one of the P*_crn-6_nuc-1*::*gfp* transgenes (*smIs195*) almost completely rescued the TUNEL defect of the *nuc-1(lf)* 4-fold embryos (Figure 4B), suggesting that in *smIs195* embryos NUC-1 was secreted and diffused into the anterior half of the embryos to affect apoptotic DNA degradation there. The strongest evidence supporting a critical role of NUC-1 secretion for its *in vivo* DNA degradation functions comes from the observations that NUC-1 expressed in pharyngeal muscle cells in the head can rescue the *nuc-1(lf)* DNA degradation defect in apoptotic cells of the posterior ventral cord, which are situated far apart, more than half of the body length, from the head region (Figure 5B), and that removal or disruption of the NUC-1 signal peptide eliminates such a long-range rescuing activity that is typical of secreted and diffusing molecules (Figure 5B). These results thus reveal a totally unexpected and previously uncharacterized mode of DNase II action: it is secreted from expressing cells and can travel a long distance and be retaken up by cells at distant sites to mediate DNA degradation.

After NUC-1 is secreted and diffuses across its expression border into other regions of the embryo, it is likely retaken up by either phagocytes or apoptotic cells. To find out in which cells NUC-1 acts to promote DNA degradation, we introduced the *smIs195* transgene (P*_crn-6_nuc-1*::*gfp*) into the *nuc-1(e1392)* mutants that were also defective in both cell corpse engulfment pathways. Surprisingly, *smIs195* displayed no discernable difference in rescuing the TUNEL defect of the *nuc-1(lf)* and *ced-6(lf); ced-2(lf); nuc-1(lf)* embryos, where cell corpse engulfment is blocked, and completely rescued the TUNEL defect of the *ced-6(lf); ced-2(lf); nuc-1(lf)* 4-fold embryos (Figure 4C). These observations indicate that NUC-1 can mediate DNA degradation inside apoptotic cells without the aid of neighboring phagocytes. However, these findings do not exclude the possibility that *C. elegans* DNase II can also act in phagocytes to promote apoptotic DNA degradation, when cell corpse engulfment proceeds normally. Furthermore, given the high degree similarity between *C. elegans* and mammalian DNase II enzymes, it is possible that mammalian DNase II may act similarly to promote DNA degradation.

## Materials and Methods

### Strains and culture conditions

*C. elegans* strains were cultured using standard procedures [41]. The N2 Bristol strain was used as the wild-type strain. The alleles used in the study were: LGIII: *crn-6(tm890)* (this study), *crn-7(ok866)* (*C. elegans* gene knockout consortium), *ced-6(n2095)* [36], *unc-119(ed3)* [42]; LGIV: *ced-2(e1752)* [26], *ced-3(n2433)* [32]; LGV: *unc-76(e911)* [43], *him-5(e1490)* [44]; LGX, *nuc-1(e1392)* [45], *axIs36* [46].

### Plasmid construction

Sequences of primers described in this section are shown in Table S3. Primers A1 and A2 were used to amplify the *nuc-1* cDNA from an N2 cDNA library. To construct the *nuc-1*::*gfp* vector, *nuc-1* coding sequence without a stop codon was PCR amplified from the *nuc-1* cDNA clone using primers A1 and A3 and inserted into the pPD95.79 vector (a gift from Andrew Fire). The *crn-7* coding sequence without a stop codon was PCR amplified using primers B1 and B2 and a *crn-7* RT-PCR product as the template. The *crn-6* coding sequence without a stop codon was amplified using primers C1 and C2 from the EST clone yk720e2 (a gift from Dr. Yuji Kohara). They were then cloned into pPD95.79 to generate *crn-7::gfp* and *crn-6::gfp* fusion vectors, respectively. All cDNAs were sequenced and confirmed to be identical to sequences reported in the Wormbase, except that the yk720e2 *crn-6* cDNA clone contains a base substitution (A322G, Ile108Val). To obtain the promoter sequences of three DNase II genes, the 5′ 1862 bp upstream region of the *crn-6* gene was PCR amplified using primers C3 and C4 and fosmid WRM0621aA08 as a template. The 5′ 3227 bp upstream region of the *nuc-1* gene was PCR amplified using primers A4 and A5 and fosmid WRM0611cG07 as a template. And the 5′ 818 bp upstream region of the *crn-7* gene was PCR amplified using primers B3 and B4 and fosmid WRM0613aA03 as a template. These promoter fragments were cloned into vector pPD122.56 to generate 4xNLS::GFP transcriptional fusions or cloned into vector pPD95.79 to generate transcriptional GFP fusions. Primers B1 and B5 were used to detect the *crn-7(ok866)* deletion by PCR and primers C5 and C6 were used to detect the *crn-6(tm890)* deletion. To construct the *nuc-1* cDNA clone without the signal peptide [NUC-1(22–375)], the region encoding NUC-1(22–375) was amplified by PCR using primers A8 and A3 and the *nuc-1* cDNA clone as a template. To construct the *nuc-1* cDNA clone with a defective signal peptide [NUC-1(L9E, I10E, F11E)] and the *nuc-1* cDNA clone encoding NUC-1(A21V, A22V), we performed quick-change mutagenesis using primers A9 and A10 and primers A11 and A12, respectively. The plasmid L3790 was used as the source of the *myo-2* promoter. To construct plasmids for ballistic bombardment, various coding regions tagged with GFP were fused with different promoters and the resulting translational fusions were then cloned into the PBSKrt2-*unc-119* bombardment vector (a gift from Dr. Min Han).

### Transgenic animals

Germline transformation was performed as described previously [47]. To study the expression pattern of CRN-6, N2 animals were injected with P*_crn-6_*4xNLS::GFP at 60 µg/ml and the pBluescript II SK vector at 40 µg/ml (as carrier DNA). To study the expression patterns of NUC-1 and CRN-7, N2 animals were injected with P*_nuc-1_*4xNLS-GFP at 40 µg/ml or P*_crn-7_*4xNLS::GFP at 60 µg/ml along with pRF4 [containing the dominant marker *rol-6(su1006)*] at 40 µg/ml as a transgenic marker. *nuc-1(e1392)* animals were injected with 60 µg/ml of P*_nuc-1_crn-7::gfp* and 40 µg/ml of P*_myo-2_gfp* (as a transgenic marker) to generate *smEx4085*. To express various NUC-1 mutants under the control of the *myo-2* promoter, *nuc-1(e1392)* animals were injected with P*_myo-2_*NUC-1 (50 µg/ml), P*_myo-2_*NUC-1(22-375)::GFP (50 µg/ml), P*_myo-2_* NUC-1(L9E, I10E, F11E) (60 µg/ml), or P*_myo-2_*NUC-1(A21V, A22V) (50 µg/ml), using P*_myo-2_gfp* (50 µg/ml) as a co-injection marker. To detect the GFP expression patterns of P*_myo-2_*NUC-1::GFP and P*_myo-2_*NUC-1(22-375)::GFP, N2 animals were injected with P*_myo-2_*NUC-1::GFP (50 µg/ml) or P*_myo-2_*NUC-1(22–375)::GFP (50 µg/ml) along with pRF4 at 50 µg/ml. Integrated transgenic lines, *smIs170*, *smIs172*, *smIs173*, *smIs175*, *smIs209–211*, *smIs187*, *smIs189*, *smIs195* and *smIs199* (Table S1), were generated by ballistic bombardment using Biolistic PDS-1000/He Particle Delivery System (Bio-Rad) [48].

### TUNEL staining and TUNEL/antibody double staining

TUNEL staining was performed as described previously [23] with minor modifications. The same fixation solution was used except that glutaraldehyde was omitted. After freezing embryos with liquid nitrogen for 1 hr and fixing the embryos at room temperature for 25 min, the embryos were washed once in 3 ml of Tris-Triton buffer (1% Triton X-100, 100 mM Tris pH 7.4) and twice in 3 ml PBST (1xPBS containing 0.5% Triton X-100). 40 µl of the labeling solution in PBST and 3 µl of enzyme solution prepared from an In Situ Cell Death Detection Kit (Roche, Switzerland) were then incubated with embryos at 37°C for 1.5 hr. The stained embryos were washed twice with 1 ml of PBST and soaked in 30 µl mounting solution containing DAPI. TUNEL staining was scored using the 100x optic on a Zeiss Axioplan 2 microscope. For TUNEL and anti-GFP antibody double staining, embryos were prepared and fixed as described above. After fixation, embryos were washed once each with the Tris-Triton buffer and PBST containing 0.1% BSA for 5 min, followed by blocking with PBST containing 1% BSA for 5 min. Rabbit anti-GFP antibody was used (1/200 dilution, a gift from Dr. Shu-Chan Hsu at Rutgers University) to stain the embryos at room temperature for 2 hrs. The embryos were then washed 3 times with PBST containing 0.1% BSA. Next, the labeling solution and the enzyme solution from the In Situ Cell Death Detection Kit and the Rhodamine-conjugated secondary antibody (1/500 dilution) were incubated with embryos at 37°C for 1 hr. After incubation, embryos were washed 3 times with PBST containing 0.1% BSA and soaked in 30 µl mounting solution containing DAPI. Anti-GFP immunostaining and TUNEL staining were scored using the 100x optic on a Zeiss Axioplan 2 microscope.

### Syto 11 staining

The vital DNA-binding dye Syto 11 (Invitrogen, Carlsbad, CA) was used to stain the condensed chromosomal DNA in the ventral cord apoptotic cells and the undigested bacteria DNA in the gut lumen [23]. *C. elegans* animals were collected from plates and washed twice with M9 buffer in a 1.5 ml microfuge tube. The animals were soaked in M9 buffer with 10 µM Syto 11 and rocked at room temperature for 1.5 hr in the dark. The animals were then washed with M9 at least once and let recover on plates seeded with OP50 at room temperature for 1 hr in the dark. After recovery, worms were washed twice with M9 and centrifuged. Three volumes of 30 mM NaN_3_ (compared with the volume of the animals) were added into the microfuge tube to anesthetize the worms, which were immediately observed by fluorescence microscopy with FITC filter. The pycnotic nuclei in the posterior ventral cord of the animals, from vulva to anus, were scored in late L1 to early L3 larvae unless otherwise noted. For the intestine staining, L3 to L4 larvae were scored.

### DNase activity assay

The DNase activity assays from *C. elegans* lysates were performed essentially as described previously [33] with minor modifications. Lysates from different *C. elegans* strains were prepared by sonication in lysis buffer (25 mM HEPES pH 7.4, 150 mM KCl, 0.2 mM DTT, 0.1 mM EDTA, 10% glycerol) containing protease inhibitors (Roche, Switzerland). After sonication, Triton X-100 was added to 1% and the lysates were incubated on ice for 30 min. Protein concentrations of the supernatants derived from the crude lysates were determined at OD_595_ using a Dye Reagent Concentrate (Bio-Rad). One µg of each worm extract was incubated with 750 ng of the pSL1190 plasmid DNA at 37°C for 10 min (in 50 mM acetic acid pH 5.0) in a 30 µl total volume. Samples were extracted using phenol: chloroform: isoamyl alcohol (25:24:1) (Sigma, St. Louis, MO) to remove proteins. 20 µl of each sample were then analyzed on a 1% agarose gel and stained with EtBr. CstF-64 was used as a loading control, based on western blot analysis of 30 µg of lysates using anti-CstF-64 antibody (a gift from Tom Blumenthal).

### Differential Interference Contrast (DIC) and fluorescence microscopy

A Zeiss Axioplan 2 microscope with a 100x optic was used to score the cell corpses in the embryos. The same 100x oil immersion optic was used with a FITC filter to score the TUNEL staining in embryos and the Syto 11 staining in larvae and with a Rhodamine filter to score the anti-GFP antibody staining in embryos. The microscope was equipped with a SenSiCam CCD camera (PCO, Germany) and Slidebook 4.0 software (Intelligent Imaging Inc. Denver, CO).

### Quantitative PCR

Quantitative PCR was performed using a Rotor-gene 3000 machine (Corbett, Australia) and SYBR Green JumpStart (Sigma, St. Louis, MO). Sequences of primers are described in Table S3. Primers C7 and C8 were used to determine the copy number of the *crn-6* gene. Primers A6 and A7 were used to determine the copy number of the *nuc-1* gene. Primers B6 and B7 were used to determine the copy number of the *crn-7* gene. The *lmn-1* gene amplified by primers D1 and D2 was used as an internal control.

## Supporting Information

Figure S1 Alignment of human and *C. elegans* DNase II genes and schematic representation of DNase II mutants. A) Sequence alignment of two human DNase II nucleases and three *C. elegans* DNase II homologues. Residues that are identical are indicated by red and residues that are similar are indicated by blue. Arrowheads indicate potential cleavage sites of signal peptides as predicted by the SignalP 3.0 program (http://www.cbs.dtu.dk/services/SignalP/), except that the cleavage site in human DNase IIα was determined experimentally. The box indicates the catalytic site of DNase II [17], [33]. Sequences of human DNase IIα (accession AAC77366) and human DNase IIβ (accession CAH73126) were used for alignment. B) Schematic representation of deletion mutations in the *crn-6* and *crn-7* genes and the *e1392* mutation in *nuc-1*. Gray boxes represent exons and waved lines indicate introns. The actual size of each deletion (*tm890* also contains an insertion) is indicated below by the black boxes.
(1.80 MB TIF)

Figure S2 Syto 11 staining of *nuc-1* and wild-type animals. DIC and Syto 11 fluorescent images of a L2 larva are shown. Arrowheads indicate pycnotic nuclei in the posterior ventral cord of the *nuc-1(e1392lf)* animal. Black arrowhead indicates the undigested bacterial DNA in the gut that is strongly stained by Syto 11. Scale bars indicate 5 µm.
(2.39 MB TIF)

Figure S3 The GFP expression patterns of P*_nuc-1_crn-6::gfp* animals. DIC and GFP images of *smIs173* transgenic animals at different developmental stages: (A) an early embryo, (B) a comma stage embryo, (C) a 2-fold stage embryo, (D) a 4-fold stage embryo, (E, F and H) larvae, and (G) adult. The head region in A–D and F are indicated by brackets. Many GFP signals were observed in the head region. In E and F, arrowheads indicate the two most anterior intestinal cells and arrows indicate the most posterior intestinal cells. Arrows in H indicates the gut lumen. The square in G indicates the vulva region. Scale bars indicate 5 µm (12.5 µm in E, F and H).
(3.68 MB TIF)

Figure S4 TUNEL assay and the GFP expression patterns of P*_crn-6_crn-6::gfp* animals. A) TUNEL analysis of *crn-6(tm890)* embryos carrying *smIs187* and *smIs189* transgenes (P*_crn-6_crn-6::gfp*). At least 30 embryos from each embryonic stage were scored. Error bars indicate SEM. (B–F) The GFP expression patterns of *smIs187* animals. DIC and GFP images of *smIs187* animals at various developmental stages are shown: B) an early embryo, C) a comma embryo, D) a 2-fold embryo, E) a 4-fold embryo, and F) a larva. Arrowheads in E and F indicate the most anterior intestinal cells. Scale bars indicate 5 µm.
(8.07 MB TIF)

Table S1 Copy number and GFP expression in various integrated lines generated by ballistic bombardment
(0.04 MB DOC)

Table S2 The crn-7 crn-6; nuc-1 triple mutant has a smaller brood size than N2 animals or any of the single mutants.
(0.03 MB DOC)

Table S3 Primers used in this study
(0.05 MB DOC)
