# Supplementary figures and images for: The Roles and Acting Mechanism of Caenorhabditis elegans DNase II Genes in Apoptotic DNA Degradation and Development
Source: PLoS One. 2009 Oct 7;4(10):e7348. doi: 10.1371/journal.pone.0007348 (PMC2752799; doi:10.1371/journal.pone.0007348)

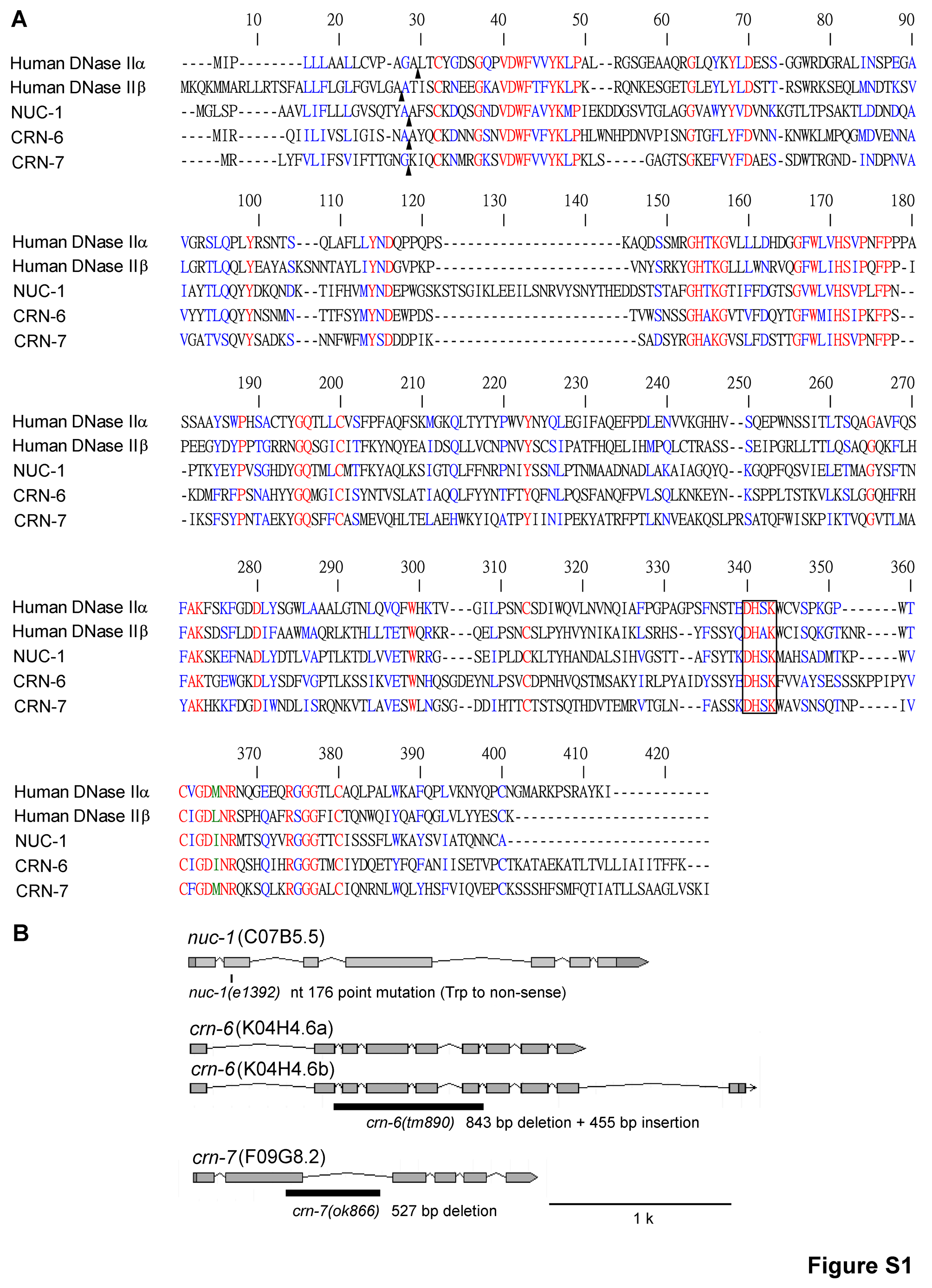

Supplement: Figure S1 — Alignment of human and C. elegans DNase II genes and schematic representation of DNase II mutants. A) Sequence alignment of two human DNase II nucleases and three C. elegans DNase II homologues. Residues that are identical are indicated by red and residues that are similar are indicated by blue. Arrowheads indicate potential cleavage sites of signal peptides as predicted by the SignalP 3.0 program (http://www.cbs.dtu.dk/services/SignalP/), except that the cleavage site in human DNase IIα was determined experimentally. The box indicates the catalytic site of DNase II [17], [33]. Sequences of human DNase IIα (accession AAC77366) and human DNase IIβ (accession CAH73126) were used for alignment. B) Schematic representation of deletion mutations in the crn-6 and crn-7 genes and the e1392 mutation in nuc-1. Gray boxes represent exons and waved lines indicate introns. The actual size of each deletion (tm890 also contains an insertion) is indicated below by the black boxes. (1.80 MB TIF) [file pone.0007348.s001.tif]

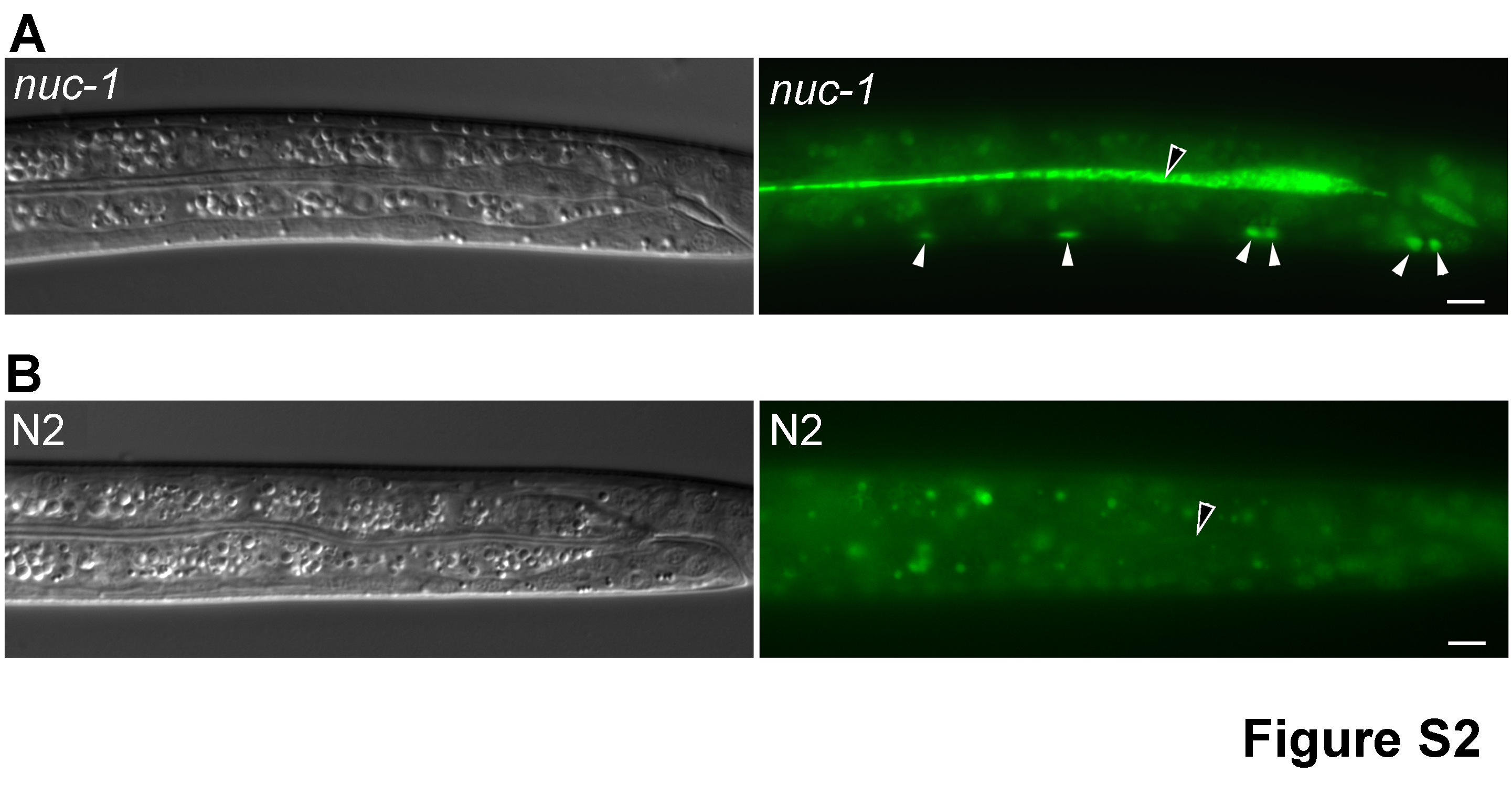

Supplement: Figure S2 — Syto 11 staining of nuc-1 and wild-type animals. DIC and Syto 11 fluorescent images of a L2 larva are shown. Arrowheads indicate pycnotic nuclei in the posterior ventral cord of the nuc-1(e1392lf) animal. Black arrowhead indicates the undigested bacterial DNA in the gut that is strongly stained by Syto 11. Scale bars indicate 5 µm. (2.39 MB TIF) [file pone.0007348.s002.tif]

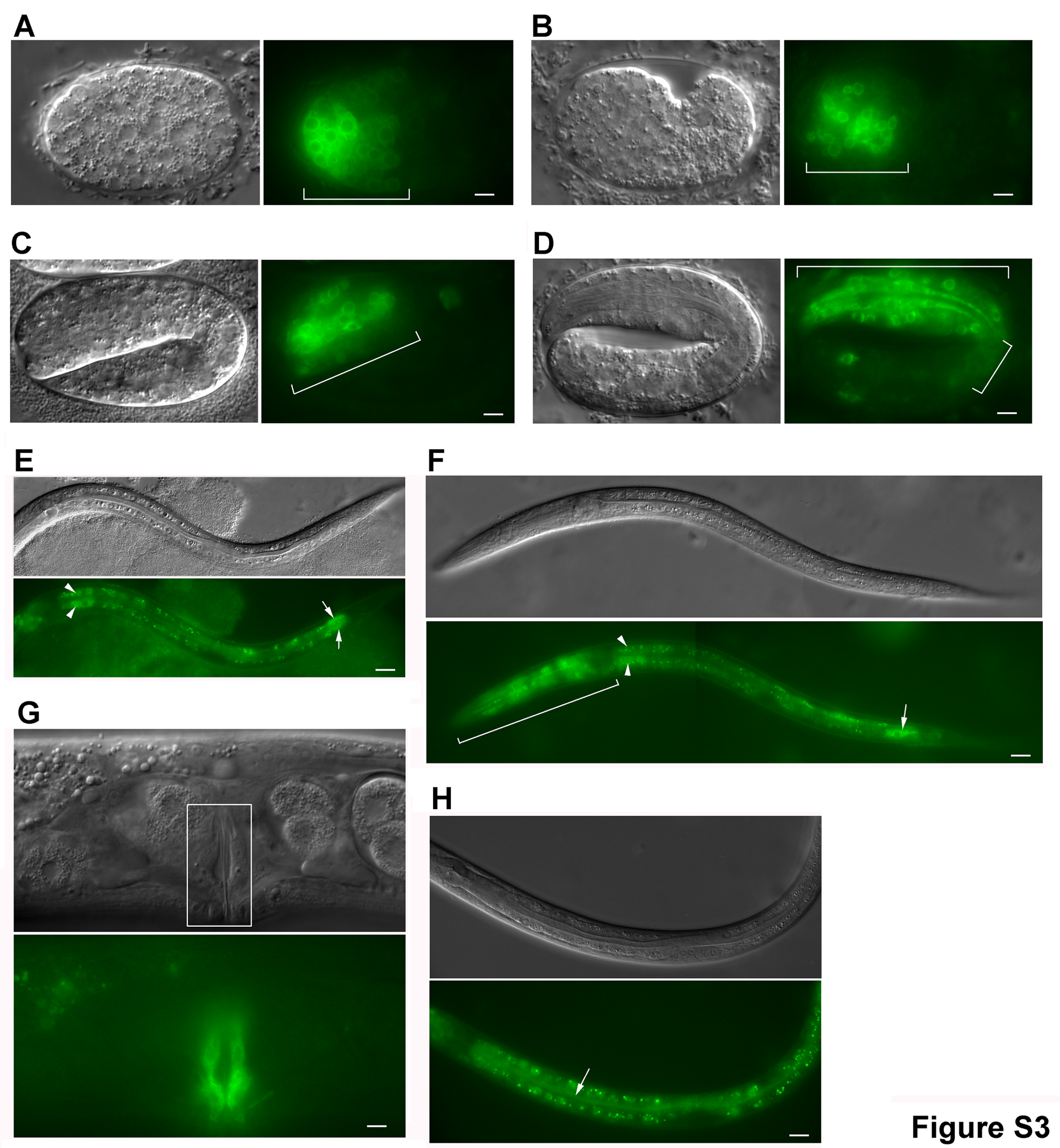

Supplement: Figure S3 — The GFP expression patterns of Pnuc-1crn-6::gfp animals. DIC and GFP images of smIs173 transgenic animals at different developmental stages: (A) an early embryo, (B) a comma stage embryo, (C) a 2-fold stage embryo, (D) a 4-fold stage embryo, (E, F and H) larvae, and (G) adult. The head region in A–D and F are indicated by brackets. Many GFP signals were observed in the head region. In E and F, arrowheads indicate the two most anterior intestinal cells and arrows indicate the most posterior intestinal cells. Arrows in H indicates the gut lumen. The square in G indicates the vulva region. Scale bars indicate 5 µm (12.5 µm in E, F and H). (3.68 MB TIF) [file pone.0007348.s003.tif]

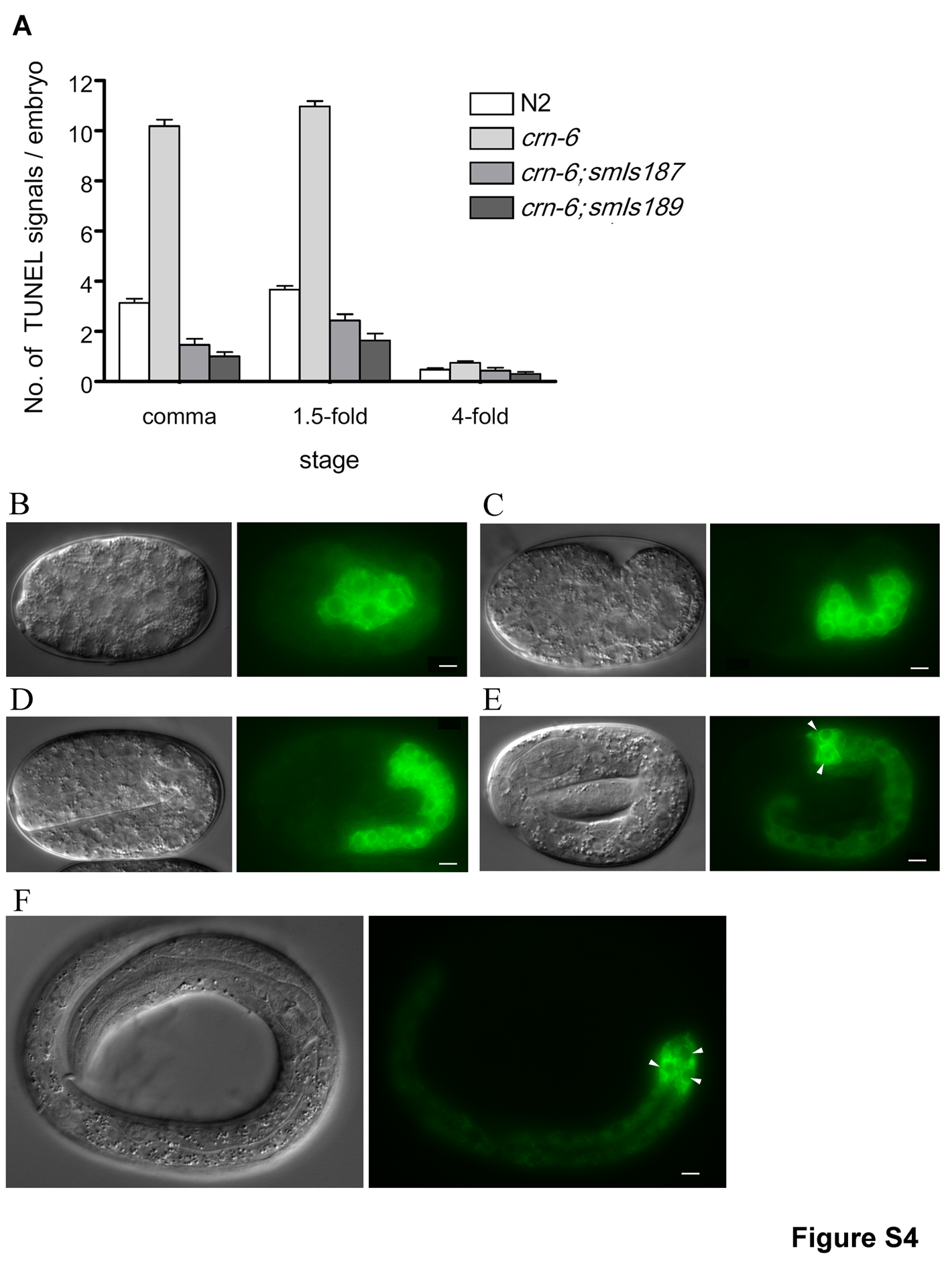

Supplement: Figure S4 — TUNEL assay and the GFP expression patterns of Pcrn-6crn-6::gfp animals. A) TUNEL analysis of crn-6(tm890) embryos carrying smIs187 and smIs189 transgenes (Pcrn-6crn-6::gfp). At least 30 embryos from each embryonic stage were scored. Error bars indicate SEM. (B–F) The GFP expression patterns of smIs187 animals. DIC and GFP images of smIs187 animals at various developmental stages are shown: B) an early embryo, C) a comma embryo, D) a 2-fold embryo, E) a 4-fold embryo, and F) a larva. Arrowheads in E and F indicate the most anterior intestinal cells. Scale bars indicate 5 µm. (8.07 MB TIF) [file pone.0007348.s004.tif]
