# Supplementary material for: The Roles and Acting Mechanism of Caenorhabditis elegans DNase II Genes in Apoptotic DNA Degradation and Development
Source: PLoS One. 2009 Oct 7;4(10):e7348. doi: 10.1371/journal.pone.0007348 (PMC2752799; doi:10.1371/journal.pone.0007348)
Supplement: Table S1 — Copy number and GFP expression in various integrated lines generated by ballistic bombardment (0.04 MB DOC) [file pone.0007348.s005.doc]

| Transgene | Expression construct | GFP signal | Copy number |
| --- | --- | --- | --- |
| *smIs170* | P*_nuc-1_nuc-1*::*gfp* | +^a^ | 3-4 |
| *smIs172* | P*_nuc-1_nuc-1*::*gfp* | +^a^ | 14-15 |
| *smIs173* | P*_nuc-1_crn-6*:: *gfp* | ++++ | 31-32 |
| *smIs175* | P*_nuc-1_crn-6*:: *gfp* | ++ | 7-8 |
| *smIs209* | P*_nuc-1_crn-7*:: *gfp* | - | 7-8 |
| *smIs210* | P*_nuc-1_crn-7*:: *gfp* | - | 3-4 |
| *smIs211* | P*_nuc-1_crn-7*:: *gfp* | - | 1-2 |
| *smIs187* | P*_crn-6_crn-6*:: *gfp* | ++++ | 29-30 |
| *smIs189* | P*_crn-6_crn-6*:: *gfp* | + | 6-7 |
| *smIs195* | P*_crn-6_nuc-1*:: *gfp* | + ^a^ | 5-6 |
| *smIs199* | P*_crn-6_nuc-1*:: *gfp* | + ^a^ | 2-3 |
| *smEx4085^b^* | P*_nuc-1_crn-7*:: *gfp* | ++++ | ND |

^a^ Weak GFP signals were observed only in the intestine of larvae.

^b^ *smEx4085* is an extrachromosomal transgenic array (see Materials and Methods for detail)
