# Supplementary material for: The Roles and Acting Mechanism of Caenorhabditis elegans DNase II Genes in Apoptotic DNA Degradation and Development
Source: PLoS One. 2009 Oct 7;4(10):e7348. doi: 10.1371/journal.pone.0007348 (PMC2752799; doi:10.1371/journal.pone.0007348)
Supplement: Table S2 — The crn-7 crn-6; nuc-1 triple mutant has a smaller brood size than N2 animals or any of the single mutants. (0.03 MB DOC) [file pone.0007348.s006.doc]

| Strain | brood size ^a^ (n) | hatching % ^a^ |
| --- | --- | --- |
| N2 (wild-type) | 256.2 ± 28.5 (10) | 99.1 ± 0.7 |
| *crn-6(tm890)* | 262.2 ± 37.0 (9) | 99.8 ± 0.3 |
| *nuc-1(e1392)* | 263.5 ± 32.1 (10) | 99.5 ± 0.5 |
| *crn-7(ok866)* | 298.0 ± 21.3 (15) | 99.6 ± 0.4 |
| *crn-6(tm890); nuc-1(e1392)* | 224.4 ± 31.6 (14) | 99.8 ± 0.2 |
| *crn-7(ok866); nuc-1(e1392)* | 244.0 ± 29.7 (13) | 99.0 ± 0.9 |
| *crn-7(ok866) crn-6(tm890)* | 240.8 ± 26.2 (14) | 99.7 ± 0.4 |
| *crn-7(ok866) crn-6(tm890); nuc-1(e1392)* | 121.2 ± 29.6 (19) | 99.2 ± 0.7 |
| *ced-3(n2433)* | 184.9 ± 24.1 (15) | 99.3 ± 0.8 |
| *crn-7(ok866) crn-6(tm890); ced-3(n2433); nuc-1(e1392)* | 110.4 ± 20.1 (13) | 99.2 ± 0.8 |

The brood size was scored as the number of eggs laid by hermaphrodite animals and the hatching % was scored as the percentage of laid eggs that hatched, which is described in detail in Materials and Methods.

^a^ Data shown are mean ± S.D.
