# Supplementary material for: The Roles and Acting Mechanism of Caenorhabditis elegans DNase II Genes in Apoptotic DNA Degradation and Development
Source: PLoS One. 2009 Oct 7;4(10):e7348. doi: 10.1371/journal.pone.0007348 (PMC2752799; doi:10.1371/journal.pone.0007348)
Supplement: Table S3 — Primers used in this study (0.05 MB DOC) [file pone.0007348.s007.doc]

| Name | Primer sequence | note |
| --- | --- | --- |
| A1 | 5’-gaaggcctatgggcttgtctcctgcc-3’ | containing a *Stu*I site |
| A2 | 5’-gacccgggttatgcacaattattttgggttg-3’ | containing a *Sma*I site & a stop codon |
| A3 | 5’-gaaggccttgcacaattattttgggttgcaa-3’ | containing a *Stu*I site |
| A4 | 5’-aactcgagttatgaatatgatagttgagatcgc-3’ | containing a *Pst*I site |
| A5 | 5’-tcccccgggttttccgctgaaattttgaaagtttc-3’ | containing a *Sma*I site |
| A6 | 5’-ggaacttctggagtctggttgg-3’ |  |
| A7 | 5’-cgctggacaataccaaaaaggac-3’ |  |
| A8 | 5’-gaaggcctatggcattctcctgcaaggatc-3’ | containing a *Stu*I site |
| A9 | 5’-cttgtctcctgccgctgtggaggaggagctcttattaggagtttc-3’ |  |
| A10 | 5’-gaaactcctaataagagctcctcctccacagcggcaggagacaag-3’ |  |
| A11 | 5’-ggagtttcccaaacatatgtagtattctcctgcaaggatc-3’ |  |
| A12 | 5’-gatccttgcaggagaatactacatatgtttgggaaactcc-3’ |  |
| B1 | 5’-gcaggcctatgcgattatattttgttcttatattt-3’ | containing a *Stu*I site |
| B2 | 5’-gaaggcctaatctttgataccaggccggc-3’ | containing a *Stu*I site |
| B3 | 5’-aactcgaggcatacataacgagacatcaattc-3’ | containing a *Pst*I site |
| B4 | 5’-tcccccgggctctcaccacctactgaattgtttc-3’ | containing a *Sma*I site |
| B5 | 5’-ctgactagtactcgtacaag-3’ |  |
| B6 | 5’-gagctagcctttccggcgctggaacatc-3’ |  |
| B7 | 5’-gactcgagccaatgctctgcgagctctg-3’ |  |
| C1 | 5’-gactcgagcaatagttgccaaggaaaccg-3’ | containing a *Stu*I site |
| C2 | 5’-gaaggcctgaaatatcgattgttggcaagc-3’ | containing a *Stu*I site |
| C3 | *5’-*aactcgaggcaaaacgttgttgattgtatttcc-3’ | containing a *Pst*I site |
| C4 | 5’-tcccccgggctgcaataatatttcagaaaaagaag-3’ | containing a *Sma*I site |
| C5 | 5’-gagcatgcatgattcgtcaaattatcttgatag-3’ | containing a *Sph*I site |
| C6 | 5’-gactcgagctagaaatatcgattgttggcaag-3’ | containing a *Xho*I site |
| C7 | 5’-gagctagccagctacatgtacaatgacgag-3’ |  |
| C8 | 5’-gactcgagcaatagttgccaaggaaaccg-3’ |  |
| D1 | 5’-cgctaagcaacaatggaggagg-3’ |  |
| D2 | 5’-gtggcaagacgactgttgagtg-3’ |  |
